# Skin microbiomes of frogs vary among body regions, revealing differences that reflect known patterns of chytrid infection

**DOI:** 10.3389/fmicb.2025.1579231

**Published:** 2025-05-13

**Authors:** Sonia L. Ghose, Jonathan A. Eisen

**Affiliations:** ^1^Genome Center, University of California, Davis, Davis, CA, United States; ^2^Department of Evolution and Ecology, University of California, Davis, Davis, CA, United States; ^3^Department of Medical Microbiology and Immunology, University of California, Davis, Davis, CA, United States

**Keywords:** microbiome, amphibian, *Rana sierrae*, skin, captivity, *Batrachochytrium dendrobatidis*, within-individual heterogeneity

## Abstract

**Introduction:**

The amphibian skin microbiome is an important line of defense against pathogens including the deadly chytrid fungus, Batrachochytrium dendrobatidis (Bd). Bd is known to preferentially infect ventral skin surfaces and feet of host amphibians, often leaving dorsal surfaces like the back uninfected. Within-individual variation in infection distribution across the skin, therefore, may relate to differences in microbiomes among skin regions. However, microbiome heterogeneity within amphibian individuals remains poorly characterized.

**Methods:**

We utilized 16S rRNA gene amplicon sequencing to compare microbiomes of 10 body regions from nine captive Rana sierrae individuals and their tank environments. These individuals were naive to Bd, allowing us to assess whether microbiomes differed among body regions prior to any impacts that may be caused by infection.

**Results:**

We found that frog skin and tank environments harbored distinct microbial communities. On frog skin, the bacterial families Burkholderiaceae (phylum Proteobacteria) and Rubritaleaceae (phylum Verrucomicrobia) were dominant, driven in large part by relative abundances of undescribed members of these families that were significantly higher on frogs than in their environment. Within individuals, we detected differences between microbiomes of body regions where Bd infection would be expected compared to regions that infrequently experience infection. Notably, putative Bd-inhibitory relative abundance was significantly higher on body regions where Bd infection is often localized.

**Discussion:**

These findings suggest that microbiomes in certain skin regions may be predisposed for interactions with Bd. Further, our results highlight the importance of considering intraindividual heterogeneities, which could provide insights relevant to predicting localized interactions with pathogens.

## 1 Introduction

Communities of microbes associated with multicellular organisms, also known as microbiomes, can play a significant role in the health and disease of their hosts (Cho and Blaser, 2012; Lee and Hase, 2014; Oever and Ten Netea, 2014; Robinson et al., 2010; Zilber-Rosenberg and Rosenberg, 2008). We are interested here in the skin-associated microbiome of frogs and the roles it may play in frog health. In general, the skin microbiome composition and structure in animals can influence host health by contributing to immune defenses and maintaining skin homeostasis (Sanford and Gallo, 2013). In amphibians, one key role of the skin microbiome is that it can serve as a primary defense mechanism against invading pathogens (Walke and Belden, 2016). The role of the amphibian skin microbiome in pathogen defense has become of great interest recently due to the global spread of the pathogen *Batrachochytrium dendrobatidis* (*Bd*), a chytrid fungus that causes the disease chytridiomycosis, which has led to dramatic declines and species extinctions in amphibians around the world (Fisher et al., 2009; Fisher and Garner, 2020; Scheele et al., 2019; Skerratt et al., 2007).

*Bd* infects keratinized epidermal cells, disrupting host osmoregulation and electrolyte balance, and often leading to mortality (Berger et al., 1998; Berger et al., 1999; Voyles et al., 2009). Interestingly, susceptibility to *Bd* varies widely among amphibian species, populations, and individuals (Jiménez and Sommer, 2017; Rosenblum et al., 2010). While this variation is influenced by multiple factors, including host genetics and environmental conditions, the skin-associated microbiome may also play a crucial role in determining susceptibility (Becker et al., 2015a). Studies have shown that several amphibian skin microbes can inhibit *Bd*, and that the structure of the skin microbiome including the number and proportion of known and/or predicted *Bd*-inhibitory taxa can predict the severity of infection and disease outcomes (Bates et al., 2018; Becker et al., 2015a, 2015b; Bell et al., 2018; Chen et al., 2022; Harris et al., 2009a; Jani et al., 2017; Woodhams et al., 2007b, 2015). These findings have spurred interest in probiotics for amphibians, although results have been mixed (Becker et al., 2009; Harris et al., 2009a, 2009b; Kueneman et al., 2016a; Becker et al., 2011, 2015a, 2021; Knapp et al., 2022; Woodhams et al., 2012, 2020). Probiotic effectiveness often depends on the ability of beneficial bacteria to persist on the skin, which is influenced by the existing microbial community (Becker et al., 2015a; Knapp et al., 2022; Woodhams et al., 2012). This underscores the need for a deeper understanding of skin microbiome complexity and dynamics in amphibians (Becker et al., 2011; Costello et al., 2012; Garner et al., 2016).

*Bd* infections in frogs are primarily limited to the ventral skin surfaces and toes (Berger et al., 1998; Berger et al., 2005; North and Alford, 2008; Pessier et al., 1999). This pattern of infection may indicate a difference in the microbial communities and niche space available in certain regions of the body, warranting an examination of the microbiomes of different body regions to understand potential regional defenses against *Bd*. Microbial heterogeneity across the skin could arise from the variability in epithelia among regions, with dorsal and ventral surfaces harboring differences in the types of glands and secretions produced (Berger et al., 2005; Varga et al., 2019). Additionally, it has been shown that bacterially produced compounds can act synergistically with host-produced anti-microbial peptides (AMPs) to inhibit *Bd* growth (Myers et al., 2012). Thus, the combination of skin architecture and bacterial composition are likely directly relevant to the distribution of *Bd* infection across the skin.

Evidence indicates that frog skin selects for specific microbes from the environment (Bates et al., 2018; Loudon et al., 2016; Walke et al., 2014), but whether there is selection for different microbes in body regions that are known to be preferentially infected by *Bd* has not been examined. In fact, few studies have assessed within-individual variability in amphibian microbiomes, although such variation has been documented in humans and other animals (Asangba et al., 2022; Bouslimani et al., 2015; Grice et al., 2009; Krog et al., 2022; Shibagaki et al., 2017; Sugden et al., 2021). Heterogeneity in microbiome structure among body regions has been detected in certain amphibian species (Bataille et al., 2016; Sabino-Pinto et al., 2016; Sanchez et al., 2017), suggesting that for at least some species, different skin regions may harbor distinct microbial communities.

In this study, we utilized high-throughput sequencing of bacterial 16S rRNA gene amplicons to characterize the skin microbiome of captive adult Sierra Nevada yellow-legged frogs (*Rana sierrae*) within individuals and their tank environments. This species has experienced dramatic population declines due to invasive fish and disease (Vredenburg et al., 2007; Vredenburg et al., 2010). Restoration efforts for this species often involve head-starting, where frogs are reared to adulthood in captivity before being reintroduced into the wild. Captivity is known to alter the amphibian skin microbiome, with several studies finding differences in microbiome structure and diversity between captive and wild individuals across many amphibian species, likely due to environmental and dietary differences (Antwis et al., 2014; Becker et al., 2014; Kueneman et al., 2022; Loudon et al., 2014; Sabino-Pinto et al., 2016). These captivity-induced shifts in the microbiome could impact the success of reintroduction programs, warranting closer attention to the microbiome in captivity (Redford et al., 2012). We were therefore interested in investigating their microbiomes in the captive setting. Further, we were interested in examining microbiomes in *Bd*-naive captive frogs so that we could determine whether frog body regions harbored differences corresponding to regions where *Bd* is expected to infect, rather than detecting differences that may have been driven by acute *Bd* infection.

By examining the skin microbiome in a captive-reared population of *R. sierrae*, we sought to address the following questions: (1) How do captive *R. sierrae* skin microbiomes differ from their tank environment microbiome? (2) How much variation is there among microbiomes of different body regions within individuals? and (3) Are there consistent differences in the skin microbiome that correspond to body regions known to be targets of *Bd* infection? We hypothesized that we would detect differences between frogs and their tank environments (Bataille et al., 2016; Walke et al., 2014) and among body regions (Bataille et al., 2016; Sabino-Pinto et al., 2016; Sanchez et al., 2017). Further, we hypothesized that certain microbes would be differentially abundant between body regions that tend to harbor *Bd* infections (ventral surfaces and feet) and body regions where infection is often absent (dorsal surfaces like the back).

## 2 Materials and methods

### 2.1 Frog population and handling

Frogs sampled for this study were members of a captive population reared to adulthood at the San Francisco Zoo from egg masses collected from a wild population in the Desolation Wilderness (El Dorado County, California; ~2,500 m elevation). This captive population was established as part of a conservation effort that was unrelated to the present study and therefore was not for research purposes alone. Adults, i.e., those with snout–vent length (SVL) ≥ 40 mm, were tagged with 8 mm unique passive integrated transponder (PIT) tags, which allow for differentiation among individuals. Groups of 8–13 frogs were housed in 52 gallon tanks (91.44 cm × 50.80 cm × 43.18 cm) filled with ~30 gallons of tap water that was filtered via reverse osmosis to remove chlorine, amines, and solids, and then supplemented with Kent Marine R/O Right, a proprietary formulation of dissolved solids and electrolytes used to restore natural water chemistry. Tanks contained biological filters to remove toxic nitrogenous compounds. We randomly selected adult frog individuals from which we collected samples and collected samples from tank environments housing those individuals.

### 2.2 Sample collection

**Figure 1:**
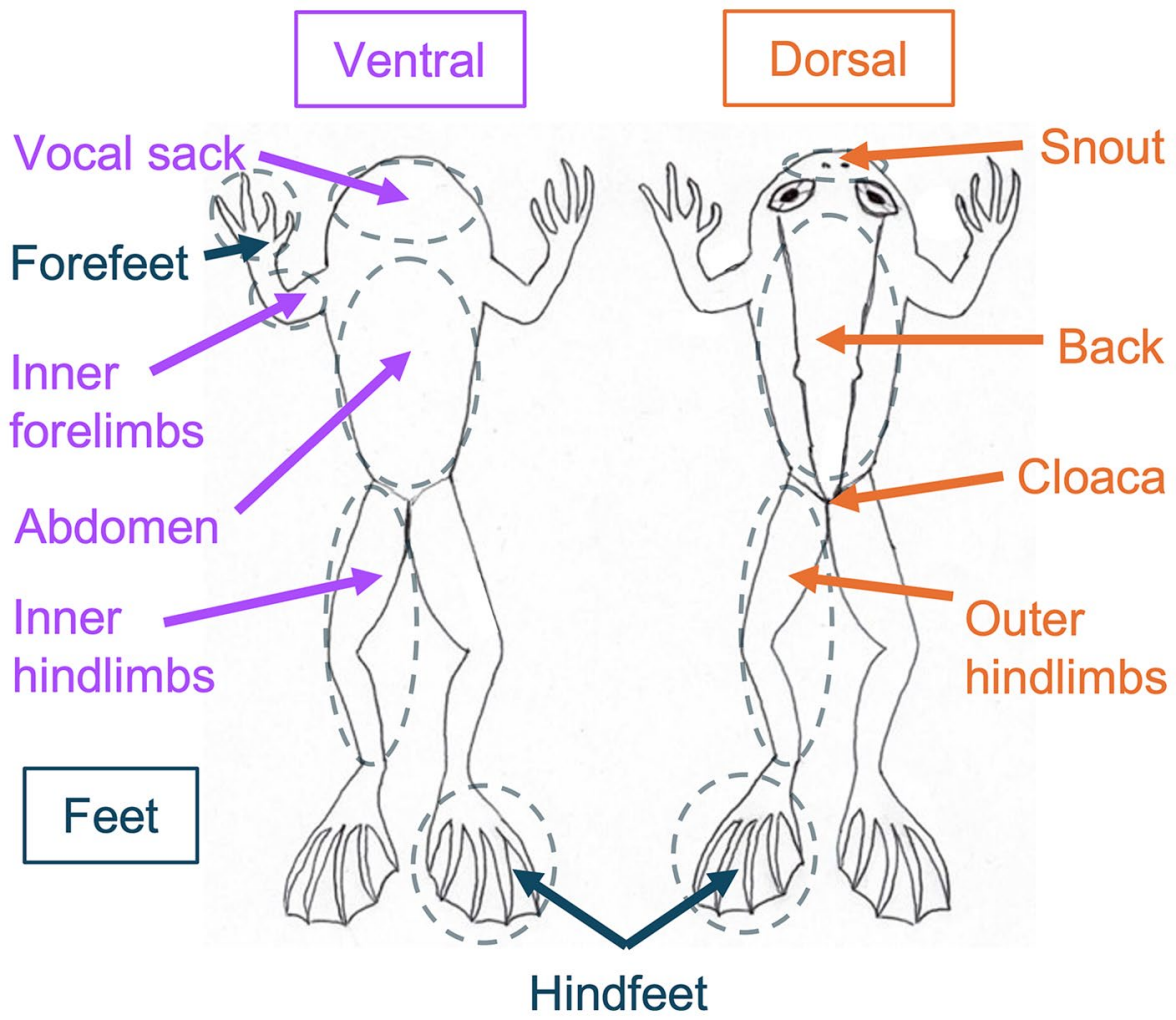
Diagram of *Rana sierrae* body regions sampled in this study. Body regions sampled are encircled in dashed lines. Ventral surfaces sampled are indicated with purple text and arrows. Dorsal surfaces sampled are indicated with orange text and arrows. Feet surfaces sampled are indicated with blue text and arrows. For limbs and feet, both the right and left were sampled. Forefeet were only sampled on the ventral surface, whereas hindfeet samples were collected from both the ventral and dorsal surfaces. Frogs sampled ranged from 46.4 to 54.4 mm snout-to-vent length (SVL) and from 9.5 to 17.8 g in weight.

We wore nitrile gloves during sample collection from frogs and surfaces in tanks using sterile synthetic fine tip dry swabs (Medical Wire & Equipment, Corsham, Wiltshire, UK; MW113). Prior to sampling, we rinsed each frog individual with 60 mL of sterile water (Culp et al., 2007; Lauer et al., 2007). From each frog, we collected a separate swab from each body region in the following order: back, outer hindlimbs, snout, vocal sack, ventral abdomen, inner forelimbs, forefeet, inner hindlimbs, hindfeet, and cloaca (Figure 1). Body regions were swabbed by taking 10 strokes to standardize sample material from regions of various sizes, taking care to only swab to the target region. The chosen target body regions were not overlapping to ensure that collected swabs were from distinct skin regions. For limbs and feet, both the left and right were sampled with the same swab. The cloaca was the smallest region sampled, for which we placed the swab under the frog vent and spun it 10 times to collect the most localized sample possible. For each frog, we also recorded the sex, tank identity, and individual identity. Next, we sampled tank environments (Supplementary Figure S1). We collected swabs of surfaces in tanks by taking 40 strokes across each surface. Surfaces sampled included (1) rock perches (affixed to the tank wall above the water surface; two samples per tank collected from the top surface of each perch), (2) underwater rocks (submerged in water; one sample per tank collected from the top surface of the rock), and (3) tank walls (three samples per tank collected from above the water surface). Tank water was sampled by filling a 60 mL syringe, passing the water through a 0.22 μm Sterivex filter (Millipore, Burlington, MA, United States), and repeating this process four times (total water filtered = 240 mL per sample; two filter samples collected per tank; Ellison et al., 2019). All samples were kept on dry ice during collection and transferred to a − 80°C freezer for storage on the same day.

In this study, we analyzed samples collected from nine frogs (*n* = 90 microbiome swabs) and their tank environments (*n* = 18 microbiome swabs; *n* = 6 water filters) on July 28, 2015. As we randomly selected frogs to sample, they were not distributed among tanks in a balanced manner: six frogs were co-housed in one tank, two frogs were co-housed in a second tank, and one frog was housed in a third tank.

### 2.3 DNA extraction

We extracted DNA from microbiome swabs and water filter samples using PowerSoil DNA Isolation Kits (MoBio Laboratories, Carlsbad, CA, United States) with a modified protocol for low-biomass samples discussed with the manufacturer. These kits have been used in several previous amphibian microbiome studies (Chen et al., 2022; Ellison et al., 2019, 2021; Kueneman et al., 2014). Swabs or filters were swirled in the PowerBead tubes and left inside these tubes. Modifications to the manufacturer’s standard protocol included the following: (1) after adding Solution C1 and vortexing to mix, tubes were incubated at 65°C for 10 min; (2) tubes were then secured in a bead beater set to “homogenize,” and bead-beated for a total of 3 min (90 s on, 60 s rest, followed by 90 s on); (3) all centrifugation steps throughout were done for 1 min at 13,000 × g unless otherwise noted below; (4) we combined steps for Solutions C2 and C3 by adding 100 μL of each at once prior to 5 min incubation on ice, (5) after the C2/C3 step, we transferred 700 μL of lysate to a clean collection tube and added 700 μL of Solution C4 and 600 μL of 100% ethanol before loading on the spin filters; (6) before washing the filter with solution C5, we inserted a step to wash with 650 μL of 100% ethanol; (7) After washing with solution C5, we dried the spin column by centrifuging for 2 min at 13,000 × g; (8) We added 60 μL of Solution C6 (heated to 60°C) to the filter membrane, and we allowed this solution to sit on the filter for 5 min before centrifuging into a storage tube. Following DNA extraction, we quantified DNA concentration using a Qubit (Invitrogen, Carlsbad, CA, United States) and the dsDNA High Sensitivity Kit, and stored DNA extracts at −80°C.

### 2.4 Sequence generation

Sequencing libraries were prepared following the protocol “16S Metagenomic Sequencing Library Preparation” (Part # 15044223 Rev. B, Illumina, Inc., San Diego, CA, United States) with some modifications. Briefly, we PCR amplified the hypervariable V3-V4 region of the bacterial 16S rRNA gene using 341F and 805R primers (Klindworth et al., 2013) with overhang adaptors (forward primer with overhang = 5′ TCGTCGGCAGCGTCAGATGTGTATAAGAGACAGCCTACGGGNGGCWGCAG; reverse primer with overhang = 5′ GTCTCGTGGGCTCGGAGATGTGTATAAGAGACAGGACTACHVGGGTATCTAATCC) from each sample in triplicate, using 4 uL DNA extract per reaction. We pooled PCR products from each sample (75 μL pool) and purified them using magnetic beads (AxyPrep Mag PCR Clean-Up Kit, Axygen, Union City, CA, United States) using 60 μL beads per pool (for a 0.8X ratio of beads to PCR product), eluting in 25 μL of 10 mM Tris pH 8.5. Next, we attached dual indices and Illumina sequencing adaptors in a second round of PCR described in the Illumina protocol. We purified and normalized 25 μL of each index PCR product using SequalPrep Normalization Plate Kits (Invitrogen, Carlsbad, CA, United States), following the manufacturer’s protocol with an extension of the binding step incubation to 2–6 h. We then pooled 10 μL of purified, normalized, and indexed PCR product per sample, and used the Zymo Clean and Concentrator Kit (Zymo Research, Irvine, CA, United States) to increase the DNA concentration of the pool following the manufacturer’s protocol (using a ratio of 5:1 of DNA Binding Buffer:PCR product, and final elution using 200 μL DNA Elution Buffer). We quantified DNA in the final pool using a Qubit (Invitrogen) and sent the pooled libraries to the UC Davis Genome Center DNA Technology Core for sequencing on an Illumina MiSeq (Illumina, Inc., San Diego, CA, United States) with v3 chemistry in 2 × 300 bp run mode.

We sequenced 20 negative control samples in addition to the biological samples. These included six controls for swab sample collection (three dry swabs and three swabs rinsed with sterile water that were placed in collection tubes at the zoo on the day of sample collections and processed in the same way as biological samples), eight blank DNA extraction kit controls (two to three preparations from each of three PowerSoil kits used to extract DNA from biological samples, for which no sample was added to the PowerBead tube but otherwise processed in the same way as biological samples), and six PCR controls (one from each time biological samples were amplified, for which no sample DNA was added to the first PCR step and subsequent processing was the same as for biological samples).

### 2.5 Sequence processing

To demultiplex the sequence data, we used a modified version of a custom script designed by G. Jospin (https://github.com/gjospin/scripts/blob/master/Demul_trim_prep.pl). Primers were removed using cutadapt v. 3.5 (Martin, 2011) with Python v. 3.9.10 (van Rossum and Drake, 2009), discarding reads for which primers were not present. We processed the resulting sequences using the DADA2 v.1.24.0 (Callahan et al., 2016) workflow in R v. 4.2.1 with RStudio v. 2022.07.0–548 (Posit Team, 2022; R Core Team, 2022). We trimmed forward and reverse reads at 250 base pairs, truncated at the first quality score of 2, and removed them if the expected errors were greater than 4 (this removed 32.8% of sequences). We then merged reads and inferred amplicon sequence variants (ASVs; 7.9% of sequences did not pass through these steps). Next, we identified 1.8% of merged reads to be chimeric and removed them. After chimera removal, samples had a mean read depth of 23,832 with a range of 294 to 98,808 reads. We assigned taxonomy to genus level using the Ribosomal Database Project (RDP) naive Bayesian classifier algorithm and the SILVA high quality ribosomal RNA database v. 132, and species level assignments were made based on exact matching of ASVs to reference strains in the SILVA database (Quast et al., 2012; Wang et al., 2007; Yilmaz et al., 2014). We then assigned unique names to ASVs, beginning with “SV” (sequence variant) followed by a number (e.g., SV1, SV2, etc.). We removed ASVs based on taxonomic classifications that were (1) non-bacterial at the domain level (including Eukaryota, Archaea, and those unclassified to domain), (2) chloroplasts, and (3) mitochondria, which resulted in 2,373 unique ASVs in the dataset.

We used Decontam v. 1.16.0 to identify putative contaminants, implementing the prevalence method with a probability threshold of 0.5 (which identifies sequences that have a higher prevalence in negative controls than in biological samples) and setting the batch argument so that contaminants were identified independently within groups of samples associated with specific negative controls (Davis et al., 2018). We identified contaminants separately for each of the following four control sample groups: (1) dry swabs (*n* = 3) setting batch by the sample material so as to identify contaminants in swab samples and not filters, (2) swabs rinsed with sterile water (*n* = 3) setting batch by whether sampling involved rinsing with sterile water so as to identify contaminants from frogs that were rinsed prior to swabbing, (3) extraction kit blanks (*n* = 8) setting batch by the PowerSoil kit used so as to identify contaminants from each kit separately, and (4) PCR negative controls (*n* = 6) without specifying batch so as to identify contaminants associated with PCR across all samples. We then compiled a list of putative contaminants identified using each control group (102 unique ASVs) and removed them, leaving 2,271 unique ASVs in the dataset.

There has been an ongoing debate in the literature regarding the validity of rarefying read counts as a sample normalization technique for microbiome data (Cameron et al., 2021; Gloor et al., 2017; McKnight et al., 2019; McMurdie and Holmes, 2014; Weiss et al., 2017). We chose to implement this method for many analyses because we were interested in community level comparisons that can become distorted using other normalization methods (McKnight et al., 2019). Additionally, rarefying was shown to be more effective than other methods at controlling effects of sample library size when sample depths are very uneven (Schloss, 2023; Weiss et al., 2017), which was the case for our dataset (read counts ranged from 2,727 to 93,792 for biological samples). We confirmed that most sample rarefaction curves plateaued at the minimum frog sample depth (i.e., 2,727 reads; Supplementary Figure S1). For all subsequent sequence analyses except for DESeq2 differential abundance testing (*see below*), samples were rarefied at an even sampling depth of 2,727. As negative control samples had fewer reads, the process of rarefying removed them from the dataset.

After rarefying the dataset, we aligned remaining sequences using DECIPHER v. 2.22.0 (Wright, 2016) and built a maximum likelihood tree with a GTR + *Γ*(4) + I model using phangorn v. 2.8.1 (Schliep et al., 2017; Schliep, 2011) on the UC Davis Bioinformatics Core High Performance Computing Cluster in R v. 4.1.0 (R Core Team, 2022). We midpoint rooted the tree using phangorn v. 2.9.0 (Schliep et al., 2017; Schliep, 2011).

The resulting dataset analyzed for this study included 1,861 unique ASVs across 114 frog and tank environment samples.

### 2.6 Microbial sequence analysis and visualization

#### 2.6.1 Alpha diversity analyses

We considered two metrics of within-sample microbial community diversity (i.e., alpha diversity): observed richness (i.e., the number of ASVs in the rarefied dataset) and Shannon diversity, which we chose to use because it incorporates both ASV richness and relative abundance (i.e., richness and evenness). We calculated these metrics using the estimate_richness function in phyloseq v. 1.40.0 (McMurdie and Holmes, 2013). Shapiro–Wilk normality tests for groups of alpha diversity estimates that we sought to compare revealed that estimates for at least one group in each comparison were not normally distributed (*p* < 0.05), warranting use of nonparametric statistical tests. We therefore implemented Kruskal-Wallis rank sum tests for significant differences in alpha diversity values between metadata groupings of the samples (including frog vs. environmental sample type and frog body region) using the kruskal.test function in base R v. 4.2.1 (R Core Team, 2022). For significant Kruskal-Wallis results (*p* ≤ 0.05), we performed *post hoc* Dunn tests with a Benjamini-Hochberg correction to control the false discovery rate (FDR) with multiple comparisons (dunnTest function in FSA v. 0.9.3; Ogle et al., 2022).

#### 2.6.2 Beta diversity analyses

To assess community structure, we compared between-sample diversity (i.e., beta diversity) using three ecological distance metrics: unweighted UniFrac, weighted UniFrac, and Bray-Curtis dissimilarities. Unweighted UniFrac distance is calculated from the community phylogenetic tree as the unique fraction of branch length within a sample community that is not shared with other communities sampled (Lozupone and Knight, 2005). This metric, therefore, can be thought of as measuring distances based on community membership (presence/absence). Weighted UniFrac takes into account the relative abundances (i.e., evenness) of branch lengths in addition to membership, giving more weight to dominant organisms than rare ones (Lozupone et al., 2007). Bray-Curtis also takes into account species richness and relative abundances, but this metric is not informed by phylogeny. We used the ordinate function in phyloseq v. 1.40.0 (McMurdie and Holmes, 2013) to calculate these distances for different subsets of the data, and visualized ordinations using principal coordinate analysis (PCoA).

To test for significant effects of metadata variables on microbial community structure, we ran permutational multivariate analysis of variance (PERMANOVA) using the adonis function in vegan v. 2.6.2 with 9,999 permutations (Anderson, 2001; Oksanen et al., 2022). For each ecological distance metric, we ran a PERMANOVA on the whole dataset (frog samples and environmental samples) to test whether significant variation in microbiome structure was explained by frog and environmental sample types. We then ran sequential PERMANOVAs on the frog samples alone to test for significant effects of frog body regions after controlling for individual and/or tank effects, as well as to test for significant effects of other metadata factors like frog sex (i.e., males versus females). Next, for factors of interest that rejected the null hypothesis in these PERMANOVAs (*p* ≤ 0.05), we performed *post hoc* pairwise PERMANOVA tests to identify which levels within factors differed significantly, using the adonis.pair function in EcolUtils v. 0.1 with 9,999 permutations (Salazar, 2022). We corrected *p*-values for multiple comparisons using the Benjamini-Hochberg procedure.

PERMANOVA tests are sensitive to differences in group dispersion (i.e., within-group variance) and thus significant effects detected by these tests could indicate differences in the average location of groups in ordination space (i.e., group centroids), differences in group dispersion, or some combination of the two (Anderson, 2001). If PERMANOVA tests indicate a significant effect of a factor and group dispersions for that factor do not differ significantly, then we know that centroid location differs for these groups. However, if the group dispersions are significantly different, then our tests are unable to distinguish whether differences are due to dispersion alone or some combination of dispersion and centroid location. We therefore also calculated mean dispersion for factors included in *post hoc* pairwise PERMANOVAs and tested for significant differences using the betadisper and permutest.betadisper functions in vegan v. 2.6.2 (Oksanen et al., 2022). For significant comparisons (*p* ≤ 0.05), we implemented *post hoc* Tukey honest significant differences tests to identify which levels within factors differed significantly in their group dispersion (using the TukeyHSD function in base R, which corrects *p*-values for the family wise error rate in multiple comparisons).

#### 2.6.3 Analyses of presence and relative abundance of taxa

We calculated the proportion of taxa shared between frog samples and environmental samples in the rarefied dataset. To do this, we first merged samples by frog and environment sample categories (i.e., collapsing read counts within each sample category), and removed any ASVs that had zero counts across frog and environmental samples. Next, we calculated the proportion of ASVs that were present in both frog and environment samples (“shared”). We also calculated the proportion of bacterial families that were shared between frog and environmental samples by collapsing ASVs from the same families using the tax_glom function in phyloseq v. 1.40.0 (specifying NArm = FALSE to include unclassified taxa; McMurdie and Holmes, 2013) prior to merging samples by frog and environment sample categories.

We visualized and compared the mean relative abundance of taxa for frog and environmental samples. We first transformed the rarefied sample counts to relative abundances and merged ASVs from the same families using the tax_glom function in phyloseq (specifying NArm = FALSE to include unclassified taxa). We grouped data by a metadata factor to compare frog samples to the four environmental sample types and by taxonomic ranks and then calculated mean relative abundances. We used microshades v. 1.11 (Dahl et al., 2022) to generate a stacked bar chart that simultaneously displays a higher taxonomic rank (phylum) and a lower taxonomic rank (family). We assigned colors to the five phyla that represented the highest relative abundance across samples and to the highest relative abundance families within those phyla, with remaining taxa represented in “Other” categories.

To determine whether the relative abundance of bacterial families or ASVs differed significantly between frog and environmental sample types, we implemented nonparametric Kruskal-Wallis rank sum tests followed by *post hoc* Dunn tests when results of Kruskal-Wallis tests were significant (*p* ≤ 0.05). We corrected *p*-values from Dunn tests using the Benjamini-Hochberg procedure.

#### 2.6.4 DESeq2 differential relative abundance testing

We used DESeq2 v. 1.36.0 in R on filtered but un-rarified merged read counts to determine which ASVs showed significant log_2_ fold differences between frog body regions (Love et al., 2014). Based on previous findings that *Bd* infection is more prominent on ventral surfaces and toes than on the dorsal back surface of many anurans (Berger et al., 1998; Berger et al., 2005; North and Alford, 2008; Pessier et al., 1999), we chose to compare frog back samples to (1) abdomen samples, (2) inner hindlimb samples, (3) hind feet samples, and (4) forefeet samples, to examine whether bacterial community members were differentially associated with these body regions.

First, we used the phyloseq_to_deseq2 function to format the raw read count data from frog samples for DESeq2. Our ASV counts table was sparse, with only two ASVs present across all samples. Therefore, to prevent geometric means and estimated size factors for DESeq2 sample normalization from being influenced solely by these ASVs, we calculated geometric means across samples for each ASV by ignoring samples with zero counts. Then, we estimated size factors for each sample based on the geometric means (applying the estimateSizeFactors function). We filtered out low relative abundance ASVs with 10 or fewer reads total across frog samples (this filtered out 1984 unique ASVs, leaving 287 unique ASVs across the frog samples). We then ran the DESeq function on the dataset for each contrast of interest, identifying ASVs that showed significant log_2_ fold differences (i.e., that showed differential relative abundance; Benjamini-Hochberg corrected *p*-values ≤ 0.05). For ASVs that showed differential relative abundance based on DESeq2 normalized counts, we calculated the mean relative abundance across body regions from the rarefied dataset. We confirmed that a nonparametric test was appropriate (Shapiro–Wilk, *p* < 0.05 for multiple body regions) and implemented Kruskal-Wallis rank sum tests to determine whether mean rarefied relative abundance also differed significantly among body regions.

#### 2.6.5 *Bd*-inhibitory predictions

We predicted putative *Bd*-inhibitory function of frog-associated microbial community members. Predictions were based on a database of full length 16S rRNA gene sequences from bacteria that were isolated and assayed for their effects on growth of *Batrachochytrium* pathogens (Woodhams et al., 2015). This database, which is regularly updated, has been used in several previous studies to predict anti-*Bd* function from amplicon data (Bletz et al., 2017; Chen et al., 2022; Jiménez et al., 2022; Kueneman et al., 2016a, 2022; Muletz Wolz et al., 2018). We used the strict inhibitory subset of the database (AmphiBac_InhibitoryStrict_2023.2; accessed from https://github.com/AmphiBac/AmphiBac-Database/) which included sequences from 2,056 inhibitory taxa. We used nucleotide BLAST v.2.9.0 to make a multiple sequence alignment with ASV sequences as queries and the inhibitory database as subject sequences, specifying a minimum *e*-value threshold of 1e−10 (Camacho et al., 2009). We documented the top hit result for cases where query coverage was 100% (i.e., cases where the alignment included the entire query ASV sequence) and the percent identity was ≥ 99% (i.e., cases where the query ASV shared ≥ 99% sequence similarity with an inhibitory database taxon). We defined these ASVs as putatively *Bd*-inhibitory. We also defined a strict subset of putative *Bd*-inhibitory taxa which included only ASVs that shared 100% sequence similarity with inhibitory database taxa.

We calculated putative *Bd*-inhibitory relative abundance in frog samples based on our strict subset of taxa (i.e., those sharing 100% sequence similarity to known inhibitory taxa) by taking the sum of read counts for the identified ASVs and dividing by the total number of reads (i.e., 2,727 reads in the rarefied dataset). To test for significant differences among frog body regions, we implemented nonparametric Kruskal-Wallis rank sum tests after finding that values were not normally distributed for multiple body regions (Shapiro–Wilk, *p* < 0.05). We then conducted *post hoc* Dunn tests, correcting *p*-values using the Benjamini-Hochberg procedure.

## 3 Results

### 3.1 Alpha diversity

#### 3.1.1 Frogs vs. tank environment microbiome diversity

**Figure 2:**
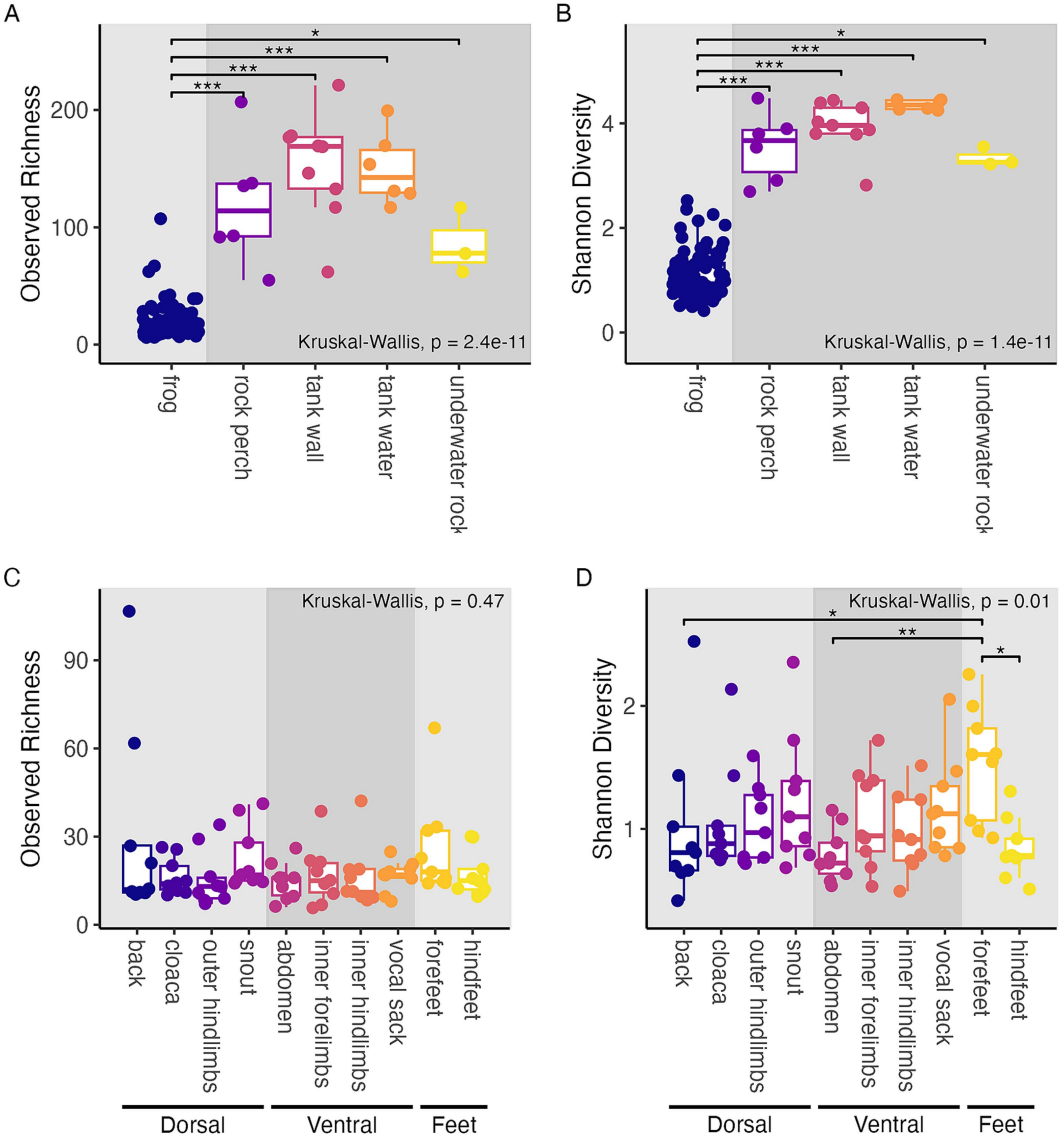
Alpha diversity-based comparisons of sample groupings. Within-sample diversity in terms of observed richness **(A,C)** and Shannon diversity **(B,D). (A,B)** Frog and tank environment samples with boxplots and points colored by the type of sample substrate. Panel background shading differentiates frog samples from tank environment samples. **(C,D)** Frog samples with boxplots and points colored by frog body region. Panel background shading differentiates groups of body regions sampled (dorsal, ventral, and feet). **(A–D)** Results of Kruskal-Wallis tests are shown. *Post hoc* Dunn test results are displayed as significance bars where applicable (*p ≤ 0.05; **p ≤ 0.01; ***p ≤ 0.001).

Within-sample diversity was significantly lower in frog samples than in tank environment sample types (including rock perch, tank wall, tank water, and underwater rock samples) based on both the observed richness (Kruskal-Wallis, chi-squared = 55.62, df = 4, *p* < 0.001; Dunn tests, *p* < 0.05; Supplementary Table S1; Figure 2A) and Shannon diversity (Kruskal-Wallis, chi-squared = 56.77, df = 4, *p* < 0.001; Dunn tests, *p* < 0.05; Supplementary Table S1; Figure 2B).

#### 3.1.2 Variation in frog microbiome diversity by body region

We found that Shannon diversity differed significantly by frog body region, (Kruskal-Wallis, chi-squared = 21.54, df = 9, *p* = 0.01; Figure 2D), but that observed richness did not (Kruskal-Wallis, chi-squared = 8.67, df = 9, *p* = 0.47; Figure 2C). Frog forefeet harbored higher Shannon diversity than the abdomen, back, and hind-feet in *post hoc* comparisons (Dunn tests, *p* < 0.05; Supplementary Table S2). In other words, while all frog body regions harbored a similar number of microbial community members, the forefeet harbored communities with higher evenness than certain other regions.

### 3.2 Beta diversity

#### 3.2.1 Frog vs. tank environment microbiome structure

**Figure 3:**
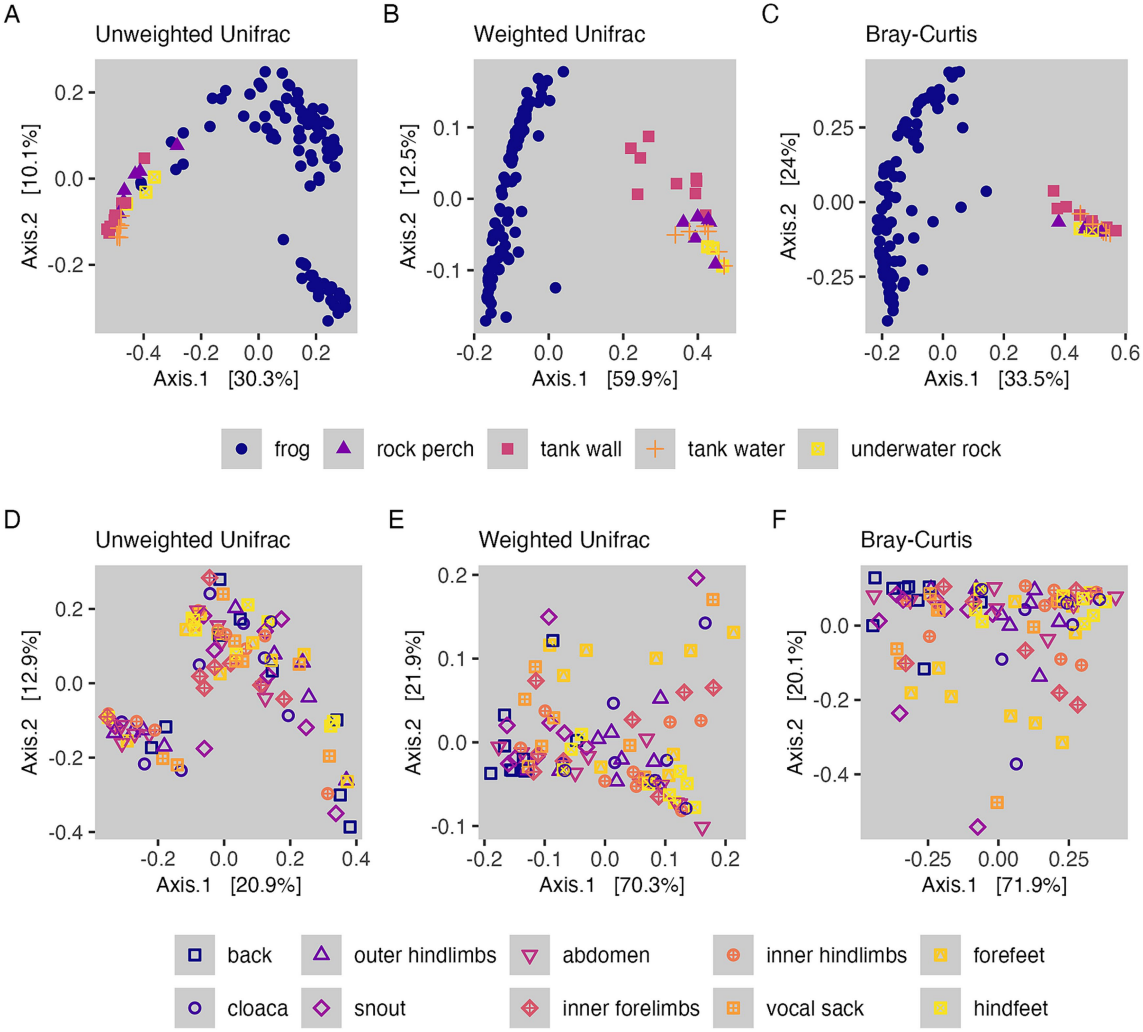
Beta diversity-based comparisons of sample groupings. **(A–C)** Microbial community structure of frog and tank environment samples with points colored and shaped by the type of sample substrate. **(D–F)** Microbial community structure of frog samples with points colored and shaped by body region. **(A,D)** PCoA visualizations of unweighted UniFrac distances. **(B,E)** PCoA visualizations of weighted UniFrac distances. **(C,F)** PCoA visualizations of Bray-Curtis dissimilarities.

Microbial community structure was significantly different between frog samples and tank environment sample types based on all three ecological distance metrics examined (PERMANOVA, *p* < 0.001; Figures 3A–C; Supplementary Table S3). *Post hoc* pairwise PERMANOVA tests revealed that all groups (frog, tank water, tank wall, rock perch, and underwater rock) were significantly different from each other based on all three metrics (PERMANOVA, *p* < 0.05; Supplementary Table S4). However, the relative importance of community characteristics measured by each metric differed. We found that differences between microbial assemblages on frogs and those from environmental samples explained the highest amount of variation in weighted UniFrac (69%), followed by Bray-Curtis (45%), and finally unweighted UniFrac (29%) (Supplementary Table S3).

We found group dispersion between frogs and environment sample types did not differ significantly for unweighted UniFrac (betadisper permutest, *p* = 0.16; Supplementary Table S5), indicating that differences based on phylogenetically informed community membership were due to differences in mean centroid locations of groups in ordination space. There were significant differences in group dispersion, however, for weighted UniFrac and Bray-Curtis (betadisper permutest, *p* < 0.001; Supplementary Table S5). For weighted UniFrac, pairwise comparisons of group dispersion (for frog, rock perch, tank wall, tank water, and underwater rock sample groupings) revealed that five out of ten comparisons were significantly different, which included two out of the four comparisons with frog samples (TukeyHSD, *p* < 0.05; Supplementary Table S6). For Bray–Curtis dissimilarity, three out of ten pairwise comparisons of group dispersion were significantly different, and these represented three of the four comparisons with frog samples (TukeyHSD, *p* < 0.05; Supplementary Tables S6, S7). However, ordination visualizations (Figures 3A–C) showed that frog samples clustered separately from environmental sample types for all three measures of community structure, which is evidence that in cases where differences in dispersion were detected between frog and environment samples, both the group dispersions and centroid locations may have been distinct.

#### 3.2.2 Drivers of frog microbiome structure

Our sequential PERMANOVA models for microbial community structure within frog samples revealed that body region explained the same amount of variation after accounting for tank identity and individual identity as it did when included as the first term (Supplementary Table S7). The interaction terms for these factors were not significant and were dropped from the models. Body region explained the highest amount of variation in Bray-Curtis (32%; *p* < 0.001), followed by weighted UniFrac (28%; *p* < 0.001), and explained the least amount of variation in unweighted UniFrac distances (11%; *p* < 0.05). We also tested whether frog sex (i.e., males versus females) explained variation in the microbiome. However, when this factor was included after individual identity in sequential PERMANOVA models, it was not significant; thus, sex was dropped from the model.

#### 3.2.3 Variation in frog microbiome structure among body regions

We used pairwise PERMANOVA tests to determine which frog body regions drove the variation in community structure explained by this factor. For unweighted UniFrac, none of the 45 pairwise comparisons of body regions were significantly different after correcting *p*-values for multiple comparisons (PERMANOVA, *p* > 0.05; Supplementary Table S8; Figure 3D). For weighted UniFrac, nine pairwise comparisons of body regions were significantly different (PERMANOVA, *p* < 0.05; Supplementary Table S8; Figure 3E), and these included seven significant differences between the back and other body regions, and two significant differences between the snout and other body regions (hindfeet and cloaca were significantly different from both the back and the snout; the abdomen, forefeet, inner forelimbs, inner hindlimbs, and outer hindlimbs were all significantly different from the back). For Bray Curtis, 13 pairwise comparisons of body regions were significantly different (PERMANOVA, *p* < 0.05; Supplementary Table S8; Figure 3F), including the same seven significant differences with the back and two significant differences with the snout that were identified for weighted UniFrac, as well as four additional significant differences with the snout only identified for Bray-Curtis (the snout also differed from the abdomen, forefeet, inner hindlimbs and outer hindlimbs for Bray-Curtis). The vocal sack was the only region that never differed significantly from other body regions in terms of community structure.

Group dispersion by body region did not differ significantly for any distance metrics (betadisper permutest, *p* > 0.05; Supplementary Table S9). These results indicate that significant results from pairwise PERMANOVA tests comparing frog body regions represented significant differences in group centroids for community structure and not differences in dispersion among body regions.

### 3.3 Presence and relative abundance of frog vs. tank environment microbial taxa

To investigate the similarity in presence of taxa between frog samples and environmental samples, we calculated the proportion of taxa shared between frog and environment. We found that 15.4% of ASVs were shared between frog and environmental samples (212 out of 1,378 ASVs). We also looked at the proportion of families shared between frog and environmental samples, which was 38.4% (91 out of 237 bacterial families).

**Figure 4:**
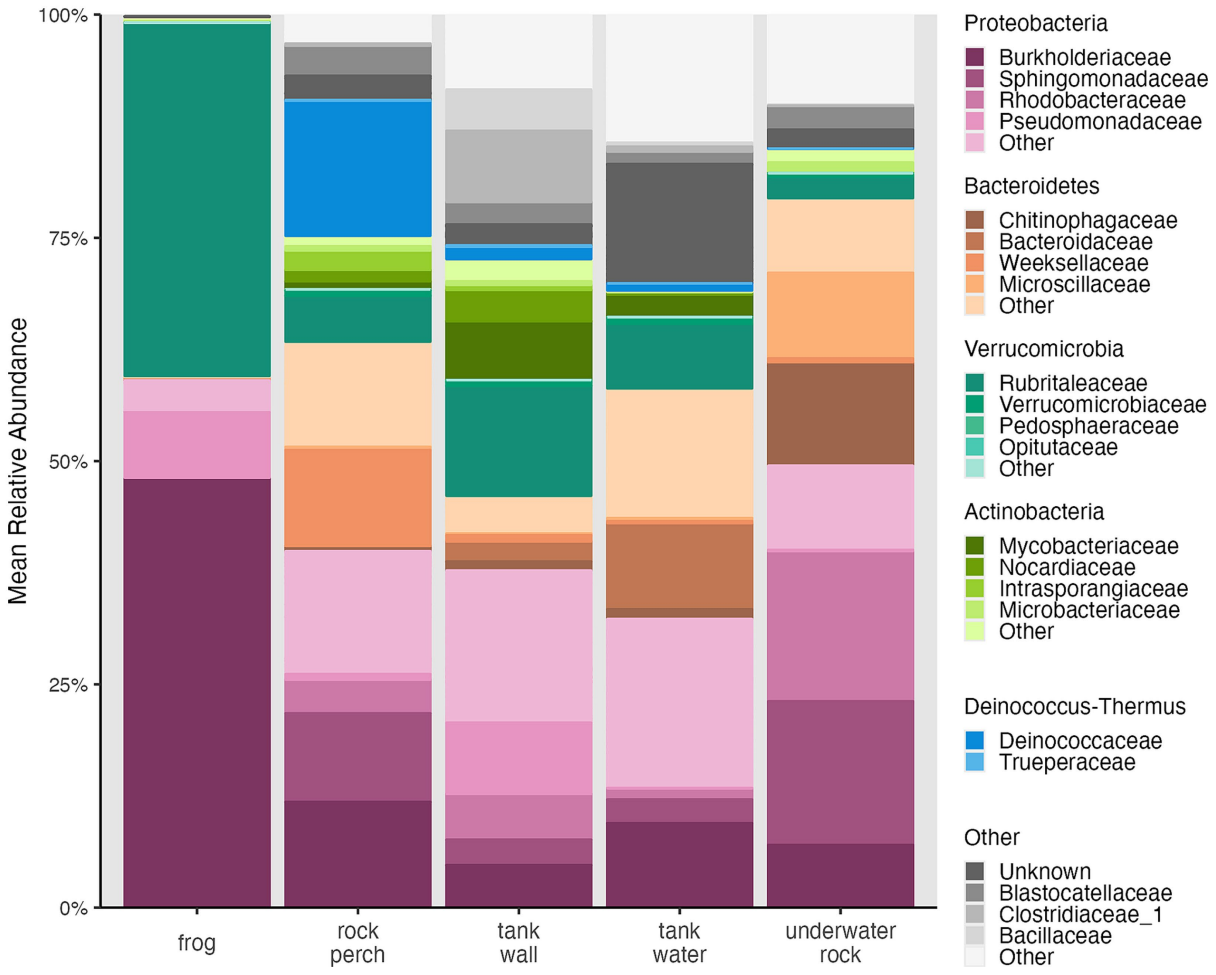
Mean relative abundances of bacterial phyla and families. Stacked bar chart for frog, rock perch, tank wall, tank water, and underwater rock samples was generated using microshades v. 1.11 (Dahl et al., 2022). Taxa are colored by phylum, and families within each phylum are colored with unique shades of the associated phylum color. Phyla and families are ordered so that higher mean relative abundance groups appear lower in stacked bars. Blastocatellaceae belong to the phylum Acidobacteria. Clostridiaceae and Bacillaceae belong to the phylum Firmicutes.

To better understand the composition of microbiomes defining different types of samples, we visualized taxonomic families across all samples. This further revealed that the distribution of taxa was distinct between frog samples and the four types of environmental samples (Figure 4). Frog-associated communities were dominated by the families Burkholderiaceae (phylum Proteobacteria; mean relative abundance of 48.0 ± 2.6% SE across frogs), Rubritaleaceae (phylum Verrucomicrobia; mean relative abundance of 39.5 ± 2.5% SE across frogs), and to a lesser extent the family Pseudomonadaceae (mean relative abundance of 7.3 ± 1.2% SE across frogs) and other families in phylum Proteobacteria. The environment-associated communities were, for the most part, made up of lower relative abundances (mean relative abundance < 17%) of an increased number of families representing several phyla in addition to the Proteobacteria and Verrucomicrobia, including the Acidobacteria, Actinobacteria, Bacteroidetes, Deinococcus-Thermus, and Firmicutes (Figure 4).

Next, we examined whether there were significant differences in relative abundances of taxa of interest between frogs and environmental sample types. Relative abundance of the family Burkholderiaceae was significantly higher on frogs than on the four environment sample types (Kruskal-Wallis, chi-squared = 357.31, df = 4, *p* < 0.001; Dunn test, *p* < 0.001; Supplementary Table S10; Figure 4). Within the Burkholderiaceae, one ASV (“SV2,” which was unclassified at the genus level) was dominant on frogs, making up 98.8% of Burkholderiaceae rarefied read counts across frog samples and 31.42% of Burkholderiaceae rarefied read counts across environmental samples. While SV2 was present in 100% of samples, its relative abundance was significantly different between frogs and environmental sample types (Kruskal-Wallis, chi-squared = 56.49, df = 4, *p* < 0.001), with significantly higher relative abundance on frogs than in the environmental sample types (Dunn tests, *p* < 0.01; Supplementary Table S11).

The family Rubritaleaceae was also dominated by one ASV present in 100% of samples (“SV1,” which was unclassified at the genus level) that made up 99.9% of Rubritaleaceae rarefied read counts across frog samples and 92.68% of Rubritaleaceae rarefied read counts across environmental samples. While the relative abundance of the family Rubritaleaceae was not significantly different between frogs and environment sample types (Kruskal-Wallis, chi-squared = 3.66, df = 4, *p* = 0.45; Figure 4), SV1 relative abundance was significantly different between these groups (Kruskal-Wallis, chi-squared = 45.62, df = 4, *p* < 0.001), with higher relative abundance on frogs than in environmental samples (Dunn tests, *p* < 0.01; Supplementary Table S11).

### 3.4 DESeq2 differential relative abundance testing among frog body regions

To identify ASVs that were differentially abundant between body regions known to experience higher *Bd* infection and pathogenesis (e.g., ventral surfaces and toes) and body regions known to have markedly lower *Bd* infection (e.g., dorsal surfaces, mainly the back) we implemented DESeq2 analysis on raw read count data. We individually compared DESeq2 normalized counts from the abdomen, the inner hindlimbs, the hindfeet, and the forefeet to the back. The analysis identified one unique ASV, an undescribed member of the family Burkholderiaceae, that showed significant log_2_ fold higher normalized counts on the abdomen compared to the back (SV56; estimated log_2_ fold difference of 24.18; Supplementary Table S12). The mean relative abundance of this ASV in the rarefied dataset, however, was not significantly different among body regions (Kruskal Wallis, chi-squared = 5.56, df = 9, *p* = 0.78; Supplementary Figure S3).

### 3.5 *Bd*-inhibitory predictions among frog body regions

We identified 73 frog-associated ASVs that were putatively *Bd*-inhibitory, based on sharing ≥ 99% sequence identity with taxa known to inhibit *Bd* growth (Woodhams et al., 2015). Assigned taxonomy for all 73 ASVs is shown in Supplementary Table S13. Our strict subset of putative *Bd*-inhibitory taxa included 29 of these ASVs that shared 100% sequence identity with inhibitory database taxa (Supplementary Table S13). The dominant undescribed Burkholderiaceae across frog samples (SV2), shared 100% sequence identity with a known *Bd*-inhibitory taxon, AmphiBac_1576 (Woodhams et al., 2015). Another undescribed Burkholderiaceae that showed significant log_2_ fold higher normalized relative abundance on the *R. sierrae* abdomen than on their back (SV56; *see above*), shared 99.53% sequence identity with the same inhibitory taxon.

**Figure 5:**
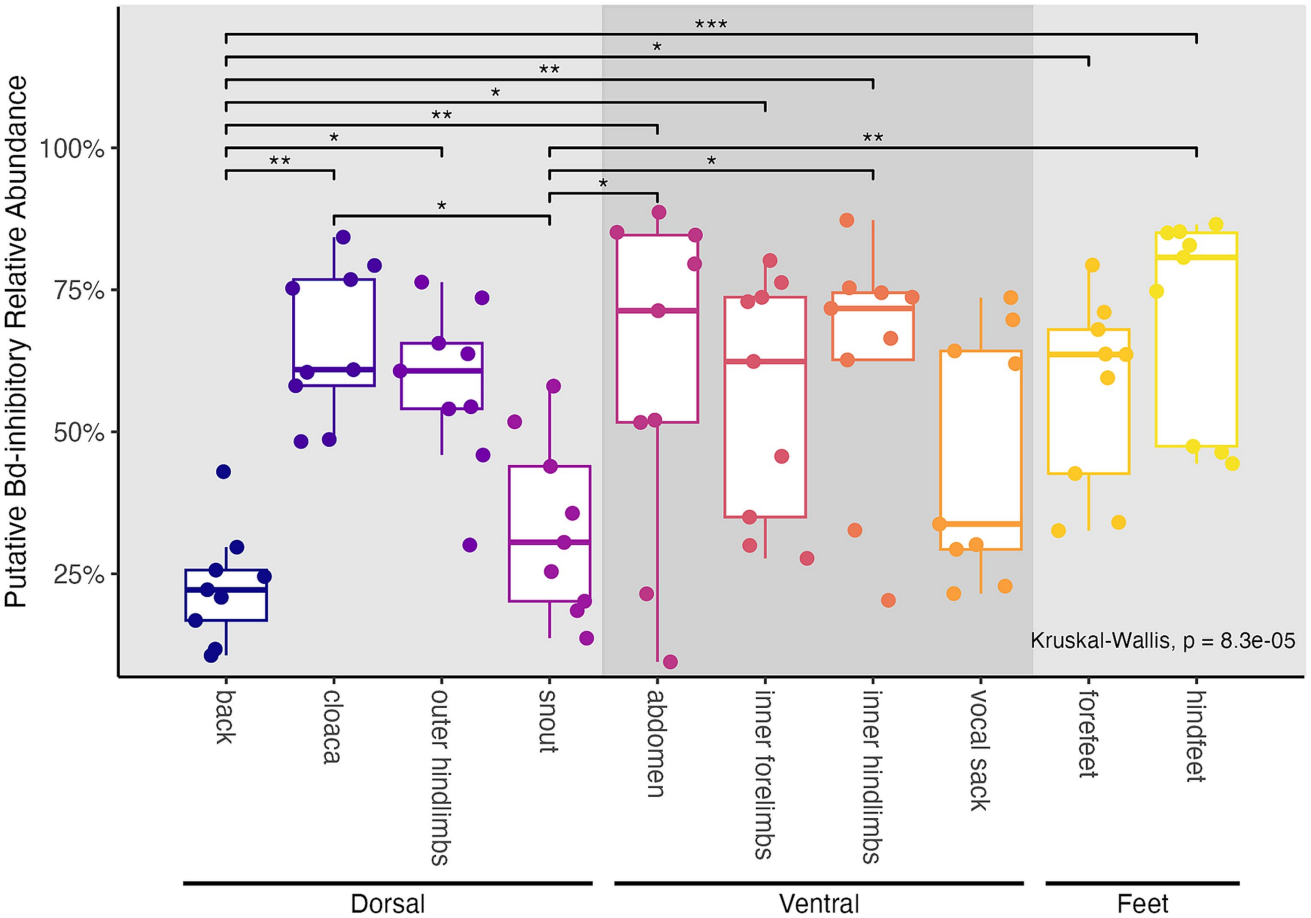
Relative abundances of putative *Bd*-inhibitory taxa among frog body regions. Values were calculated based on our strict subset of 29 frog-associated ASVs that shared 100% sequence identity with taxa known to inhibit *Bd* growth (Woodhams et al., 2015). Boxplots and points are colored by frog body region. Panel background shading differentiates groups of body regions sampled (dorsal, ventral, and feet). Results of Kruskal-Wallis tests are shown. *Post hoc* Dunn test results are displayed as significance bars where applicable (*p ≤ 0.05; **p ≤ 0.01; ***p ≤ 0.001).

Total putative *Bd*-inhibitory relative abundance for our strict subset of ASVs (i.e., 29 ASVs that had identical sequences to inhibitory database taxa) differed significantly by frog body region (Kruskal-Wallis, chi-squared = 34.19, df = 9, *p* < 0.001; Figure 5). The back harbored significantly lower relative abundance of putative *Bd*-inhibitory taxa than seven other body regions (the abdomen, cloaca, forefeet, hindfeet, inner forelimbs, inner hindlimbs, and outer hindlimbs; Dunn tests, *p* < 0.05; Supplementary Table S14) and the snout harbored significantly lower relative abundance of putative *Bd*-inhibitory taxa than four other body regions (the abdomen, cloaca, hindfeet and inner hindlimbs; Dunn tests, *p* < 0.05; Supplementary Table S14). We confirmed that results were similar for ASVs sharing ≥ 99% similarity with inhibitory database taxa.

## 4 Discussion

Our fine-scale analysis of the skin microbiome identified characteristics that vary between captive frogs and their tank environments, and among body regions within individuals. We found that frogs harbored distinct microbial communities compared to their local tank environment. In addition, there were detectable differences between microbiomes of body regions known to be targets of *Bd* infection compared to those regions that infrequently experience infection. Further, elevated relative abundances of putatively *Bd*-inhibitory microbes were localized in body regions where we would expect interactions with *Bd* to occur. Together, these results help elucidate the captive microbiome of the endangered Sierra Nevada yellow-legged frog, *R. sierrae*, and provide a basis for predicting microbiome-pathogen interactions.

### 4.1 Frog skin microbiomes were distinct from their tank environment microbiome and were dominated by fewer organisms

We hypothesized that frog skin microbiomes would be distinct from their surrounding tank environment microbiome, which was supported by our results for within-sample community diversity (i.e., alpha diversity), community structure (i.e., beta diversity), and presence and relative abundance of microbial community members. We found that tank substrates and water harbored significantly higher within-sample diversity than frogs (Figures 2A,B), which agrees with a previous study of wild *R. sierrae* showing that lake water communities had higher observed richness than frog associated communities (Ellison et al., 2019). However, this finding differed from previous evidence that lake water microbiomes had reduced or equal diversity compared to microbial communities associated with several other species of post-metamorphic amphibians (Bates et al., 2018; Kueneman et al., 2014). The discrepancy between our results here and those of these prior studies has multiple possible explanations. One possibility is that lake water collected by Ellison et al. (2019) and tank water collected here were unusually diverse compared to other environments. Another possibility (and we note, both could be occurring) is that *R. sierrae* may harbor lower diversity microbiomes than other species. Reduced diversity is linked to clinical signs of chytridiomycosis (Becker and Harris, 2010) while higher community richness has been shown to correlate with host persistence after *Bd* invasion (Jani et al., 2017). Therefore, if *R. sierrae* microbiomes harbor lower diversity and richness than other species, this could relate to their high susceptibility to *Bd* (Vredenburg et al., 2010). Additional studies that directly compare the diversity of *R. sierrae* microbiomes to other species would be useful here.

We also found that community structure significantly differed between frog-associated and environment-associated microbiomes (Figures 3A–C), which has been previously reported in studies of both captive and wild amphibian populations (Albecker et al., 2019; Bates et al., 2018; Fitzpatrick and Allison, 2014; Jani et al., 2017; Jani and Briggs, 2014; Kueneman et al., 2014; Walke et al., 2014). We note that the ordinations comparing frog and environmental samples showed a characteristic “horseshoe effect,” which could be a consequence of saturation of the distance metrics (Morton et al., 2017). Since distance metrics cannot discriminate between samples that do not share ASVs, this saturation can occur when the dataset has sparse counts of ASVs across samples creating a “band table” (Morton et al., 2017). This effect does not alter our interpretation of the ordinations, and it actually provides further evidence of the high dissimilarity between environment and frog samples. There are several factors that make amphibian skin a unique and complex environment that could lead to such differences. Mucosal secretions, anti-microbial peptides (AMPs), and other secretions produced by the host regulate microbial presence and abundance on the skin, as do other microbes and the anti-microbial metabolites they produce, all of which vary between and within host species (Lillywhite and Licht, 1975; Myers et al., 2012; Tennessen et al., 2009; Walke et al., 2014; Woodhams et al., 2006a, 2006b, 2007a, 2010). By affecting which microbes can exist and persist on the skin, these interacting skin components act as a filter for microbes from the environment.

Previous studies found variable proportions of taxa shared between amphibian-and environment-associated communities, and usually dominant microorganisms on amphibians were different from those in environmental assemblages (Bates et al., 2018; Kueneman et al., 2014; Walke et al., 2014). Further, abundant microorganisms on amphibians have been shown to be rare in the environment (Bates et al., 2018; Kueneman et al., 2014; Walke et al., 2014). Here, we found that only 15.4% of ASVs were shared between frogs and environmental samples. We also looked at the proportion of shared bacterial families between frog and environmental samples, which was higher at 38.4%. We note that the proportion of taxa shared with their environment may be lower for captive frogs than their wild counterparts, as was shown previously (Bataille et al., 2016).

Additionally, in our study, a major difference in the distribution of taxa between frogs and their tank environments was that the two sequence variants found to be dominant on frogs (SV1 in the family Rubritaleaceae and SV2 in the family Burkholderiaceae) both showed significantly lower relative abundance on tank and perch substrates and in tank water. Similarly, abundance-weighted metrics for microbial community structure explained a higher proportion of variation between frogs and environmental sample types than did an unweighted metric, supporting that relative abundances of community members better define differences than community membership alone. These results may suggest that high relative abundances of certain bacteria, including the two dominant taxa on frogs, were selected for by their skin (Loudon et al., 2016; Walke et al., 2014). A caveat of these statistical comparisons is that the data used is compositional (i.e., relative abundances must sum to 100%). Therefore, care must be taken with the interpretation of differences in relative abundances across samples since they do not represent absolute abundances and are standardized to the rarefied read count. For example, the higher relative abundances of these two taxa on frogs than in their environment could have resulted from higher absolute abundances on frogs, but it also could have resulted from reduced abundances of other taxa on frogs that inflated the relative abundance of these two ASVs. Regardless, it is interesting that microbial relative abundances on *R. sierrae* skin were dominated by only two sequence variants, and this result was consistent with previous studies of both wild and captive amphibians that reported dominance by one or few bacterial strains (Bates et al., 2018; Kueneman et al., 2014, 2016b; Loudon et al., 2014).

The Rubritaleaceae are a little studied family of Gram-negative bacteria in the phylum Verrucomicrobia, containing only five described species isolated from marine animals or marine sediment (Kasai et al., 2007; Rosenberg, 2014; Scheuermayer et al., 2006; Yoon et al., 2007; Yoon et al., 2008). The 16S rRNA genes of these species are very highly conserved and the species are not distinguishable by 16S amplicon analysis (Rosenberg, 2014). This may explain why we were unable to assign taxonomy below the family level for the dominant Rubritaleaceae sequence variant on frogs. Described Rubritaleaceae species are non-motile, obligate aerobes that synthesize carotenoid pigments, resulting in red-colored colonies (Rosenberg, 2014). The production of carotenoid pigments by Rubritaleaceae on frog skin may affect the skin-associated microbial community, as previous studies have shown that dietary carotenoid intake by amphibians increased community richness and shifted community structure of frog skin microbiomes (Antwis et al., 2014; Edwards et al., 2017).

The only previous mention of the Rubritaleaceae in amphibian microbiomes was from a study of captive *Rana muscosa*, the sister species to *R. sierrae*, conducted in the same facility at the San Francisco Zoo as our study (Jani et al., 2021). However, the phylum Verrucomicrobia, which includes Rubritaleaceae, has been detected on amphibian skin in several studies based on 16S rRNA gene data (Becker et al., 2014; Belden et al., 2015; Kueneman et al., 2014, 2016b; Longo et al., 2015; Loudon et al., 2014; Sabino-Pinto et al., 2016; Sanchez et al., 2017). Verrucomicrobia is a widely distributed phylum that has been found in various environments including soil, marine and freshwater, and animal intestines (Bergmann et al., 2011; Hugenholtz et al., 1998; Parveen et al., 2013; van Passel et al., 2011a; van Passel et al., 2011b). Although a previous study found that Verrucomicrobia were higher in relative abundance on wild than captive Panamanian golden frogs (*Atelopus zeteki*; Becker et al., 2014), we hypothesize that the high relative abundance of Rubritaleaceae and Verrucomicrobia observed in the present study may be unique to captivity. This owes to the fact that Verrucomicrobia, though present, were not high in relative abundance in previous studies of wild *R. sierrae* populations (Jani and Briggs, 2014), even for populations from the same site used to source the San Francisco Zoo population examined here using the same primers for 16S rRNA gene amplification (Ellison et al., 2019, 2021). It is possible that in captivity, these taxa replace other taxa with similar functional abilities on the skin in the wild, but this requires further investigation.

The other dominant amphibian associated sequence variant was a member of the family Burkholderiaceae. This family consists of ecologically, phenotypically, and metabolically diverse Gram-negative bacteria found in soil, water, and in association with plants, animals, and fungi (Coenye, 2014). While some Burkholderiaceae are pathogens to plants and animals including humans (Coenye, 2014), others have been shown to suppress fungal pathogens (Carrión et al., 2018). The dominant Burkholderiaceae sequence variant observed here shared 100% sequence identity with a bacterial isolate from the *Bd* inhibitory database, suggesting that this bacterium may help suppress *Bd* proliferation on the skin (AmphiBac_1576/Ranamuscosa-inhibitory_37; Supplementary Table S13; Woodhams et al., 2015).

The order Burkholderiales, which includes the family Burkholderiaceae, has been identified as highly relatively abundant on amphibians in several studies (Bataille et al., 2016; Bates et al., 2018; Kueneman et al., 2014), including studies of *R. sierrae* (Ellison et al., 2019, 2021). Previous research on wild *R. sierrae* found Burkholderiaceae on their skin, but it showed lower relative abundance compared to another family in the order, Comamonadaceae (Ellison et al., 2019). Notably, several amphibian microbiome studies report that a single Comamonadaceae sequence variant dominated the community in much the same way as the dominant Burkholderiaceae sequence variant did here (Bates et al., 2018; Kueneman et al., 2014, 2016b). Interestingly, while the taxonomic assignment for this bacterium was to Burkholderiaceae, the *Bd* inhibitory bacterial isolate with identical amplicon sequence was classified as an undescribed Comamonadaceae in the inhibitory database metadata (Woodhams et al., 2015). This discrepancy illustrates how choice of assignment algorithm and/or taxonomic database can impact such classifications. Therefore, the dominant sequence variant we identified as a Burkholderiaceae is likely to be closely related to the dominant Comamonadaceae found in other amphibian studies, or it may even represent the same bacterium. Despite amplicon sequence similarity to a known *Bd*-inhibitor, further work is needed to determine whether the specific taxon identified here exhibits anti-*Bd* function.

### 4.2 Frog body regions showed spatial variation in the microbiome, which corresponded to expected spatial variation in *Bd* infection

We tested our hypotheses that we would detect differences in microbiomes among body regions within frog individuals and that some variation would correspond to body regions where *Bd* infection would be expected to occur. We examined uninfected frogs (see Supplementary material) in order to determine whether they natively harbored such differences, rather than describing *Bd*-driven impacts to microbiomes. While our findings for within-sample diversity provide some support for differences among body regions, findings for community structure and relative abundance of putative *Bd*-inhibitory taxa showed stronger support for heterogeneity among body regions and that differences were associated with predicted *Bd* localization, discussed below.

Within-sample diversity, for the most part, did not differ between frog body regions; the only exceptions were significantly higher relative Shannon diversity on frog forefeet compared to the abdomen, hindfeet, and back (Figures 2C,D). This differs from previous studies of the *Bombina orientalis* microbiome that found ventral surfaces harbored higher richness and diversity than dorsal surfaces (Bataille et al., 2016; Sabino-Pinto et al., 2016) but is consistent with findings from other amphibian species that showed no such differences (for *Bufo japonicus, Cynops pyrrhogaster, Odorrana splendida,* and *Rana japonica*; Sabino-Pinto et al., 2016). Interestingly, there did not appear to be a relationship between the size of each body part and microbiome diversity. The forefeet were one of the smallest body regions sampled, but harbored higher diversity than larger body regions like the back. This suggests that standardizing the number of strokes of each body region was sufficient to control for differences in the area occupied by each body region.

Microbial community structure differed primarily between the back and other body regions. Previous studies also found significant differences in microbiome structure across the skin for two different amphibian species (Bataille et al., 2016; Sabino-Pinto et al., 2016; Sanchez et al., 2017). Though in one study, community structure only differed significantly between dorsal and ventral surfaces in captivity and not in the wild, warranting future investigation of within-individual microbiome heterogeneity of wild *R. sierrae* (Bataille et al., 2016). Here, while we did not detect differences in unweighted community membership among body regions, we found that the back differed significantly from seven other body regions based on two metrics of relative abundance weighted community structure (Figures 3D–F). This suggests that shifts in relative abundances of community members are more important to differences between body regions than presence/absence of organisms. Supporting this claim, we found that the relative abundance of putative *Bd*-inhibitory taxa differed significantly in the same comparisons of the back with other body regions (Figure 5; Woodhams et al., 2015). These taxa had significantly higher relative abundance on body regions including the abdomen, inner hind-limbs, and feet compared to the back. In addition, one putatively *Bd*-inhibitory undescribed member of the Burkholderiaceae showed significant log_2_ fold higher normalized read counts on the abdomen compared to the back (SV56; Supplementary Tables S12, S13; Woodhams et al., 2015).

Our finding that much of what defines heterogeneity in the skin microbiome are differences between the back and other body regions supports our hypothesis that microbiome variation corresponds to spatial heterogeneity in *Bd* infection across the skin. Studies have shown that *Bd* infection occurs most on ventral surfaces, hindfeet, and toes, and is either absent or minimal (i.e., very few *Bd* sporangia) on dorsal surfaces like the back (Berger et al., 1998; Berger et al., 2005; North and Alford, 2008; Pessier et al., 1999). In addition, it has been shown that the back of an amphibian experiences fewer pathological changes due to *Bd* infection than do other body surfaces (Berger et al., 2005). The fact that putatively *Bd*-inhibitory taxa showed higher relative abundance on ventral surfaces and feet compared to the back suggests that they may directly interact with *Bd* upon infection. Further, they may help to control infection on body regions where *Bd* is known to infect. Indeed, previous work has shown that higher putative *Bd*-inhibitory relative abundance correlates with lower *Bd* loads (Chen et al., 2022). However, isolation and functional characterization of these *R. sierrae* associated taxa are needed to determine if they would act to inhibit *Bd* growth in practice. Additionally, as discussed above, the data is compositional, and therefore quantitative analyses of taxa are needed to determine whether differences in relative abundance of putatively inhibitory taxa were driven by differences in their absolute abundances or by differences in abundances of other taxa.

The reasons that both *Bd* infections and microbiomes differ among body regions may relate to differences in skin architecture of the amphibian host. For example, there are usually larger and more numerous granular glands (also referred to as serous glands) on the back compared to ventral surfaces (Berger et al., 2005; Varga et al., 2019). Granular glands secrete bioactive molecules that assist in host defense, including AMPs (Varga et al., 2019). Such differences in the skin landscape may contribute to lower *Bd* infection on the back and to the differences in the skin microbiome between the back and other body regions that we observed here.

Our results suggest that where you collect an amphibian skin swab from (i.e., which body regions) will affect the resultant community observed. However, we emphasize that this may not apply to all types of amphibians. The heterogeneity in skin structure, microbiome structure, and *Bd* localization across the skin are all likely related to the evolved ecology of the amphibian. *R. sierrae* is a semi-aquatic species that spends much of its time basking at the edges of lakes and streams, keeping ventral surfaces and toes more moist than the back. *Bd* zoospores require water to disperse, so differences in moisture across the skin due to an amphibian’s lifestyle and ecology may contribute to spatial heterogeneity of *Bd* infection across the skin. For example, in a fully aquatic amphibian species (*Xenopus tropicalus*), no differences were detected in *Bd* infection between dorsal and ventral regions (Parker et al., 2002). Future studies would benefit from comparing differences in the microbiome structure, skin architecture, and spatial heterogeneity in *Bd* infection across amphibians of differing ecologies to determine whether there are consistent patterns and to elucidate the role that ecology has played in the evolution of such differences.

## 5 Conclusion

We found that captive *R. sierrae* microbiomes were distinct from microbiomes of their local environment and that there was variation in microbiomes among different regions of their skin. Some differences across the skin aligned with regions where *Bd* is known to infect, despite the fact that sampled frogs were naive to *Bd*. We suggest that understanding microbiome variation within captive individuals could be important to interventions designed to protect amphibians threatened by *Bd*, which are often applied in the captive setting. For example, differences across the skin could be exploited to focus on altering microbiomes of ventral surfaces and feet that gain higher *Bd* loads, perhaps by boosting abundances of *Bd*-inhibitory microbes that occur natively in these skin regions. Additionally, by elucidating microbiome variation within individuals, we can better understand and develop models to predict corresponding variation in *Bd* intensity. Future studies that examine variation within wild individuals would also be useful for this purpose. Detected natural variation may relate to how susceptible frogs will be to high levels of infection (Ellison et al., 2019; Jani and Briggs, 2014), which could also be an indicator of how healthy and resilient frogs are in the face of other pathogens or environmental stressors.

## Data Availability

The raw 16S rRNA gene amplicon sequence data for this project has been deposited in the National Center for Biotechnology Information Sequence Read Archive under BioProject PRJNA1219149.
