# Supplementary material for: Skin microbiomes of frogs vary among body regions, revealing differences that reflect known patterns of chytrid infection
Source: Front Microbiol. 2025 May 13;16:1579231. doi: 10.3389/fmicb.2025.1579231 (PMC12106533; doi:10.3389/fmicb.2025.1579231)
Supplement: Supplementary file 1 [file Table_1.DOCX]

Supplementary Material

# Supplementary Methods and Results

## Assessing *Bd* infection

After collecting microbiome swab samples, an additional swab sample was collected per individual to assess *Bd* presence and infection intensity (“*Bd* swabs”). To collect *Bd* swabs, we used a standard protocol, taking 30 strokes of ventral surfaces (10 strokes of ventral abdomen, 10 strokes of inner hindlimbs, and 5 strokes on each hindfoot; (“Chytrid Swabbing Protocol,” 2009).

DNA was extracted from *Bd* swab samples using the PrepMan Ultra Sample Preparation Reagent following the manufacturer’s protocol (40 μL used per sample). DNA extracts were stored at -20 °C or -80 °C. To confirm that frogs reared at the Zoo were not infected with *Bd*, we used a standard quantitative PCR (qPCR) assay with *Bd*-specific primers for ITS1 (Boyle et al., 2004; Hyatt et al., 2007). qPCR assays of DNA extracts were run at the Sierra Nevada Aquatic Research Laboratory (Mammoth Lakes, California, USA). Plasmid *Bd* standards were used in assays, which are based on single ITS1 PCR amplicons (Joseph and Knapp, 2018; Longo et al., 2013).

No amplification of *Bd* ITS1 was detected in frog *Bd* swab samples, indicating that *Bd* was not present on frogs sampled in this study.

## References

Boyle, D.G., Boyle, D.B., Olsen, V., Morgan, J.A.T., Hyatt, a D., 2004. Rapid quantitative detection of chytridiomycosis (*Batrachochytrium dendrobatidis*) in amphibian samples using real-time Taqman PCR assay. Dis. Aquat. Organ. 60, 141–148. https://doi.org/10.3354/dao060141

Chytrid Swabbing Protocol [WWW Document], 2009. . AmphibiaWeb. URL https://amphibiaweb.org/chytrid/swab_protocol.html (accessed 11.30.23).

Hyatt, A.D., Boyle, D.G., Olsen, V., Boyle, D.B., Berger, L., Obendorf, D., Dalton, A., Kriger, K., Hero, M., Hines, H., Phillott, R., Campbell, R., Marantelli, G., Gleason, F., Colling, A., 2007. Diagnostic assays and sampling protocols for the detection of *Batrachochytrium dendrobatidis*. Dis. Aquat. Organ. 73, 175–192. https://doi.org/10.3354/dao073175

Joseph, M.B., Knapp, R.A., 2018. Disease and climate effects on individuals drive post-reintroduction population dynamics of an endangered amphibian. Ecosphere 9, e02499. https://doi.org/10.1002/ecs2.2499

Longo, A.V., Rodriguez, D., Leite, D. da S., Toledo, L.F., Almeralla, C.M., Burrowes, P.A., Zamudio, K.R., 2013. ITS1 copy number varies among *Batrachochytrium dendrobatidis* strains: Implications for qPCR estimates of infection intensity from field-collected amphibian skin swabs. PLOS ONE 8, e59499. https://doi.org/10.1371/journal.pone.0059499

# Supplementary Figures


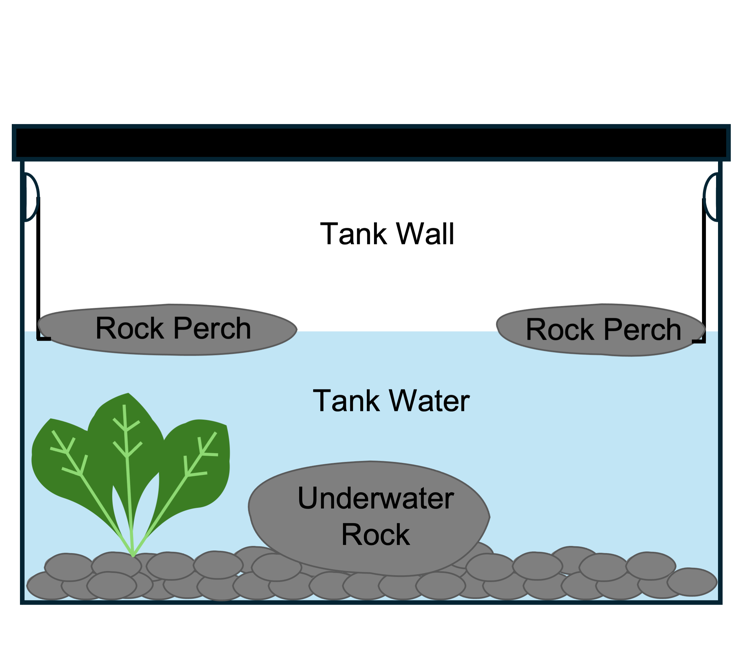


**Figure S1. Diagram of tank substrates sampled in this study.** Substrates labeled in the diagram were sampled. Each tank had two perches made of rock that were affixed to the tank wall so that they sat above the water surface (“rock perch”) and one large rock submerged under the water surface (“underwater rock”). Swab samples were collected from the top surface of each rock perch (n = 2 per tank) and of underwater rocks (n = 1 per tank). Swab samples were collected from tank walls (n = 3 per tank) above the surface of the water. Tank water was sampled by passing 240 mL through a 0.22 μm filter. Diagram dimensions are not to scale.


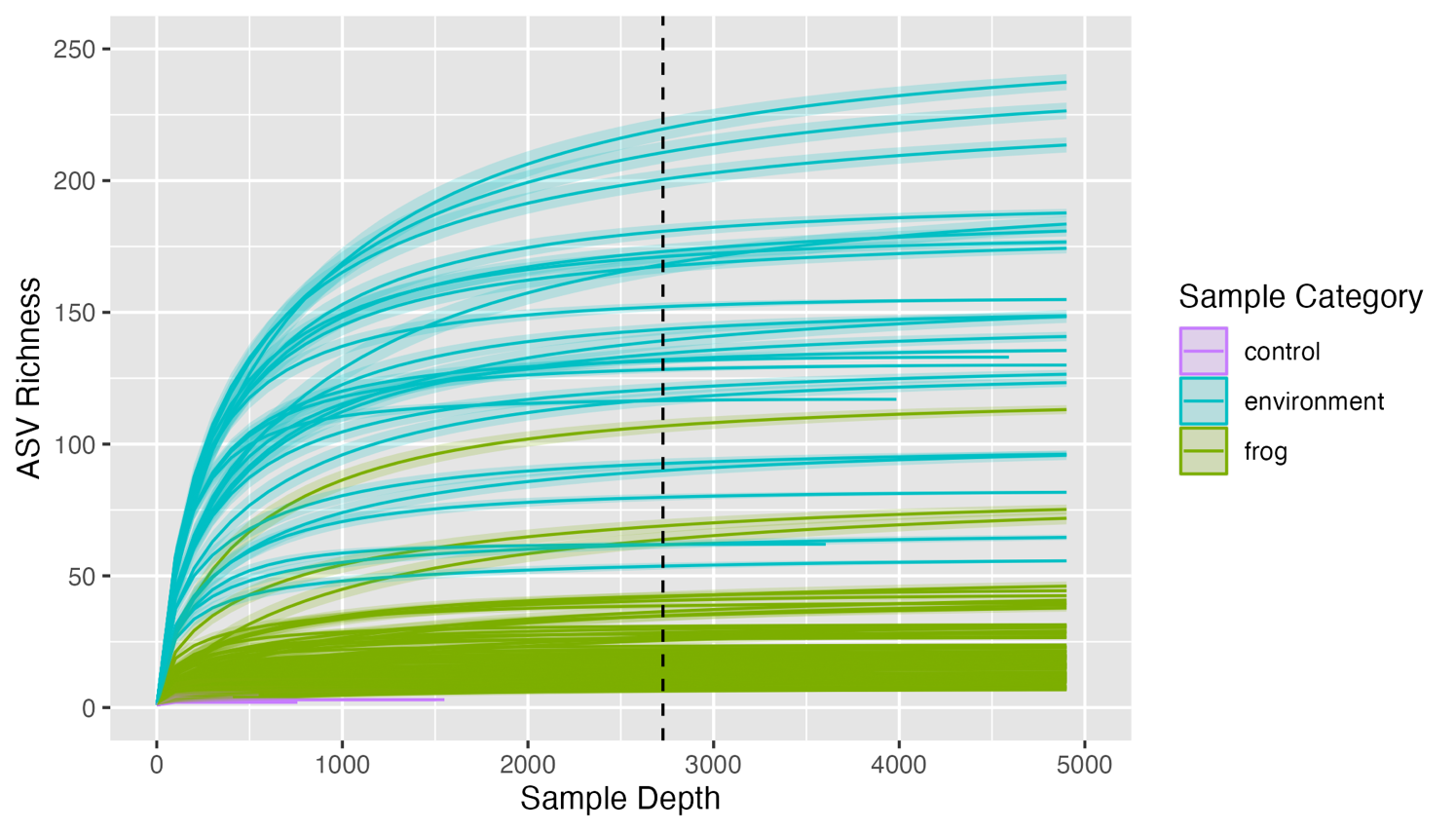


**Figure S2.** **Rarefaction curves across sample categories.** Microbiome sample curves are colored by sample categories including control samples, tank environment samples, and frog samples. Vertical dashed line shows cut-off of 2,727 reads used to rarefy to an even sampling depth. This read depth was the minimum depth of a biological sample and was chosen to maintain samples in the dataset while ensuring most samples’ ASV richness had plateaued.


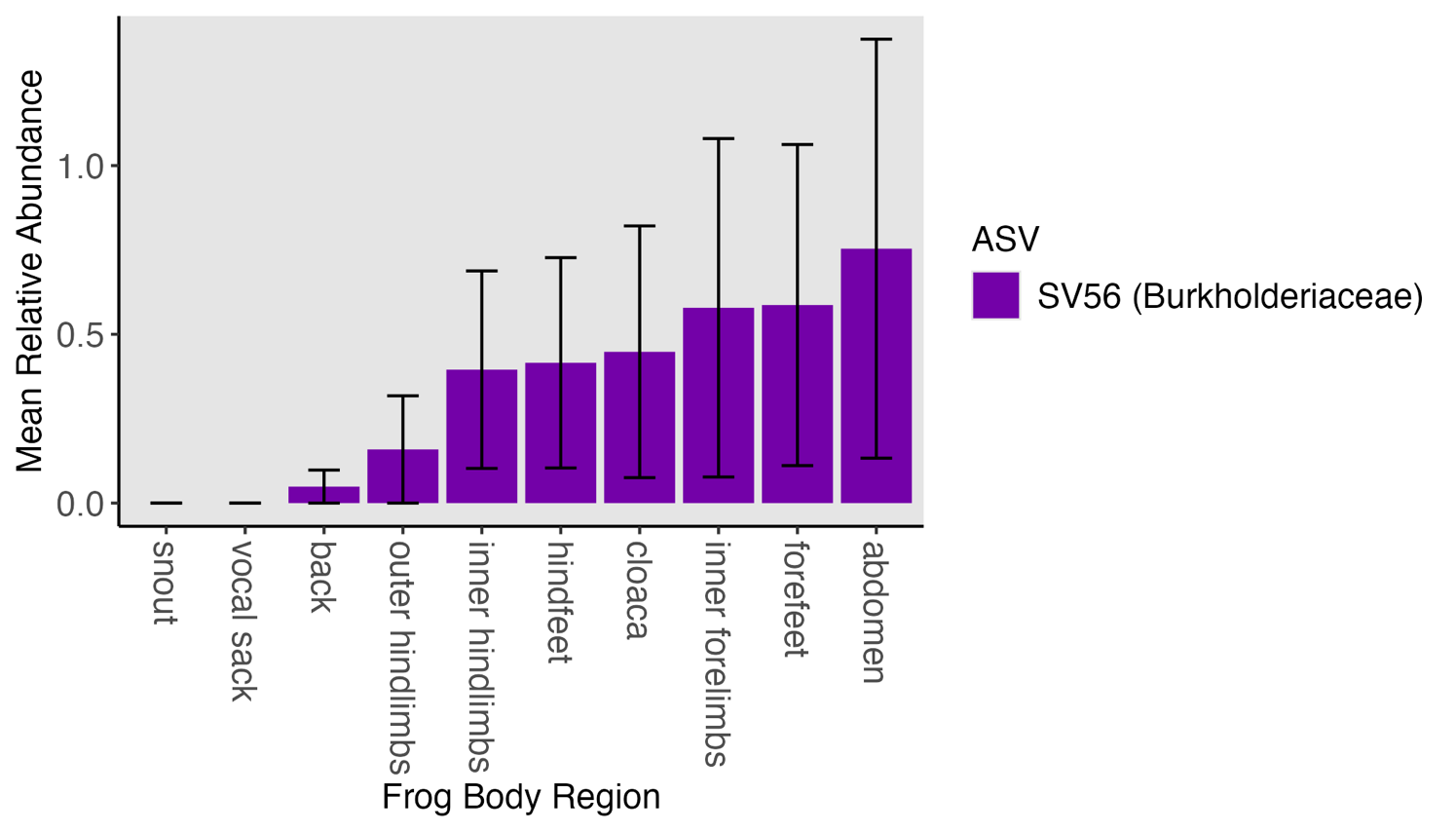


**Figure S3. Mean relative abundance of Burkholderiaceae taxon identified to have differential normalized counts between body regions.** SV56 had a significant log_2_ fold difference in DESeq2 normalized read counts between the abdomen and back (estimate of log_2_ fold difference = 24.18, p < 0.05; Table S12). Plot shows mean relative abundance of SV56 (family Burkholderiaceae) on frogs across all body regions calculated from the rarefied dataset. Frog body regions are ordered from lowest to highest mean relative abundance of SV56. Standard errors of means are represented with error bars. Mean relative abundance of SV56 was not significantly different between body regions (Kruskal Wallis, chi-squared = 5.5597, df = 9, p = 0.783).

# Supplementary Tables

**Table S1. Pairwise comparisons of alpha diversity estimates among frog and environment sample types.** Following significant results of Kruskal-Wallis tests (Shannon: chi-squared = 56.771, df = 4, p < 0.001; Observed: chi-squared = 55.624, df = 4, p < 0.001), results of *post hoc* Dunn tests for Shannon diversity and observed richness in pairwise comparisons of frog, rock perch, tank wall, tank water, and underwater rock samples are shown. P-values were adjusted using Benjamini-Hochberg procedure to control the false discovery rate (FDR) with multiple comparisons. For significance, “***” = p ≤ 0.001, “**” = p ≤ 0.01, “*” = p ≤ 0.05, and “ns” = not significant.

|  | **Comparison** | **Z** | **Unadjusted  P-value** | **Adjusted  P-value** | **Significance** |
| --- | --- | --- | --- | --- | --- |
| **Shannon** | frog ↔ rock perch | -3.851 | 0.00011771 | 0.00039238 | *** |
|  | frog ↔ tank wall | -4.966 | 0.00000068 | 0.00000682 | *** |
|  | frog ↔ tank water | -4.544 | 0.00000551 | 0.00002754 | *** |
|  | frog ↔ underwater rock | -2.569 | 0.01020177 | 0.02550442 | * |
|  | rock perch ↔ tank wall | -0.214 | 0.83080469 | 0.92311632 | ns |
|  | rock perch ↔ tank water | -0.507 | 0.61246706 | 1.00000000 | ns |
|  | rock perch ↔ underwater rock | 0.164 | 0.86972014 | 0.86972014 | ns |
|  | tank wall ↔ tank water | -0.341 | 0.73292772 | 0.91615965 | ns |
|  | tank wall ↔ underwater rock | 0.343 | 0.73168590 | 1.00000000 | ns |
|  | tank water ↔ underwater rock | 0.578 | 0.56352445 | 1.00000000 | ns |
| **Observed** | frog ↔ rock perch | -3.869 | 0.00010925 | 0.00036417 | *** |
|  | frog ↔ tank wall | -5.143 | 0.00000027 | 0.00000271 | *** |
|  | frog ↔ tank water | -4.216 | 0.00002485 | 0.00012425 | *** |
|  | frog ↔ underwater rock | -2.479 | 0.01318713 | 0.03296782 | * |
|  | rock perch ↔ tank wall | -0.316 | 0.75202298 | 1.00000000 | ns |
|  | rock perch ↔ tank water | -0.253 | 0.79989743 | 0.99987179 | ns |
|  | rock perch ↔ underwater rock | 0.250 | 0.80275295 | 0.89194772 | ns |
|  | tank wall ↔ tank water | 0.038 | 0.96944868 | 0.96944868 | ns |
|  | tank wall ↔ underwater rock | 0.515 | 0.60673714 | 1.00000000 | ns |
|  | tank water ↔ underwater rock | 0.457 | 0.64784982 | 1.00000000 | ns |

**Table S2. Pairwise comparisons of Shannon diversity among frog body regions.** Following significant results of Kruskal-Wallis test for differences in Shannon diversity (Shannon diversity: chi-squared = 21.543, df = 9, p = 0.01; Observed richness not significantly different: chi-squared = 8.674, df = 9, p = 0.468), results of *post hoc* Dunn tests for Shannon diversity in pairwise comparisons of frog body regions are shown. P-values were adjusted using Benjamini-Hochberg procedure to control the false discovery rate (FDR) with multiple comparisons. For significance, “***” = p ≤ 0.001, “**” = p ≤ 0.01, “*” = p ≤ 0.05, and “ns” = not significant.

|  | **Comparison** | **Z** | **Unadjusted  P-value** | **Adjusted  P-value** | **Significance** |
| --- | --- | --- | --- | --- | --- |
| **Shannon** | abdomen ↔ forefeet | -3.762 | 0.00016839 | 0.00757741 | ** |
|  | forefeet ↔ hindfeet | 3.320 | 0.00089963 | 0.02024164 | * |
|  | back ↔ forefeet | -2.941 | 0.00326904 | 0.04903565 | * |
|  | forefeet ↔ inner hindlimbs | 2.589 | 0.00961514 | 0.10817030 | ns |
|  | abdomen ↔ snout | -2.382 | 0.01722542 | 0.12919061 | ns |
|  | abdomen ↔ vocal sack | -2.445 | 0.01448457 | 0.13036113 | ns |
|  | cloaca ↔ forefeet | -2.156 | 0.03105981 | 0.17471141 | ns |
|  | forefeet ↔ outer hindlimbs | 2.174 | 0.02967886 | 0.19079265 | ns |
|  | hindfeet ↔ vocal sack | -2.003 | 0.04518499 | 0.22592495 | ns |
|  | forefeet ↔ inner forelimbs | 1.877 | 0.06057054 | 0.22713953 | ns |
|  | hindfeet ↔ snout | -1.940 | 0.05240730 | 0.23583285 | ns |
|  | abdomen ↔ inner forelimbs | -1.886 | 0.05934353 | 0.24276898 | ns |
|  | back ↔ snout | -1.561 | 0.11856139 | 0.33345391 | ns |
|  | abdomen ↔ outer hindlimbs | -1.588 | 0.11230735 | 0.33692205 | ns |
|  | abdomen ↔ cloaca | -1.606 | 0.10828457 | 0.34805755 | ns |
|  | back ↔ vocal sack | -1.624 | 0.10437669 | 0.36130392 | ns |
|  | hindfeet ↔ inner forelimbs | -1.444 | 0.14886506 | 0.39405458 | ns |
|  | forefeet ↔ snout | 1.380 | 0.16746460 | 0.41866149 | ns |
|  | forefeet ↔ vocal sack | 1.317 | 0.18775782 | 0.44468957 | ns |
|  | inner hindlimbs ↔ vocal sack | -1.272 | 0.20332689 | 0.45748551 | ns |
|  | hindfeet ↔ outer hindlimbs | -1.146 | 0.25186992 | 0.47225610 | ns |
|  | cloaca ↔ hindfeet | 1.164 | 0.24447925 | 0.47832896 | ns |
|  | inner hindlimbs ↔ snout | -1.209 | 0.22667256 | 0.48572691 | ns |
|  | abdomen ↔ inner hindlimbs | -1.173 | 0.24084156 | 0.49263046 | ns |
|  | back ↔ inner forelimbs | -1.065 | 0.28704814 | 0.51668665 | ns |
|  | back ↔ outer hindlimbs | -0.767 | 0.44314874 | 0.62317791 | ns |
|  | inner forelimbs ↔ inner hindlimbs | 0.713 | 0.47599815 | 0.62999755 | ns |
|  | hindfeet ↔ inner hindlimbs | -0.731 | 0.46490246 | 0.63395789 | ns |
|  | cloaca ↔ snout | -0.776 | 0.43780263 | 0.63551995 | ns |
|  | back ↔ cloaca | -0.785 | 0.43249382 | 0.64874073 | ns |
|  | abdomen ↔ back | -0.821 | 0.41163480 | 0.66155592 | ns |
|  | outer hindlimbs ↔ snout | -0.794 | 0.42722248 | 0.66293143 | ns |
|  | cloaca ↔ vocal sack | -0.839 | 0.40143309 | 0.66905516 | ns |
|  | outer hindlimbs ↔ vocal sack | -0.857 | 0.39138468 | 0.67739656 | ns |
|  | inner forelimbs ↔ vocal sack | -0.559 | 0.57590489 | 0.74044915 | ns |
|  | inner forelimbs ↔ snout | -0.496 | 0.61973852 | 0.77467315 | ns |
|  | inner hindlimbs ↔ outer hindlimbs | -0.415 | 0.67812641 | 0.78245354 | ns |
|  | cloaca ↔ inner hindlimbs | 0.433 | 0.66496714 | 0.78746108 | ns |
|  | back ↔ hindfeet | 0.379 | 0.70473808 | 0.79283034 | ns |
|  | back ↔ inner hindlimbs | -0.352 | 0.72493893 | 0.79566468 | ns |
|  | abdomen ↔ hindfeet | -0.442 | 0.65842570 | 0.80078801 | ns |
|  | cloaca ↔ inner forelimbs | -0.280 | 0.77971675 | 0.81598264 | ns |
|  | inner forelimbs ↔ outer hindlimbs | 0.298 | 0.76590728 | 0.82061494 | ns |
|  | snout ↔ vocal sack | -0.063 | 0.94964276 | 0.97122555 | ns |
|  | cloaca ↔ outer hindlimbs | 0.018 | 0.98560343 | 0.98560343 | ns |

**Table S3. PERMANOVA model for microbial community structure explained by frog and environment sample types.** Permutational multivariate analysis of variance (PERMANOVA) model outputs based on unweighted UniFrac distances, weighted UniFrac distances, and Bray-Curtis dissimilarities are shown. “EnvironType_FrogCategory” groupings include frog, rock perch, tank wall, tank water, and underwater rock samples.

|  | **Factor** | **Df** | **Sum of squares** | **Mean squares** | **F Model** | **R^2^** | **Pr(>F)** |
| --- | --- | --- | --- | --- | --- | --- | --- |
| **Unweighted UniFrac** | EnvironType_FrogCategory | 4 | 8.471 | 2.118 | 11.171 | 0.291 | 0.0001 |
|  | Residuals | 109 | 20.664 | 0.190 |  | 0.709 |  |
|  | Total | 113 | 29.136 |  |  | 1.000 |  |
| **Weighted UniFrac** | EnvironType_FrogCategory | 4 | 5.564 | 1.391 | 62.000 | 0.695 | 0.0001 |
|  | Residuals | 109 | 2.446 | 0.022 |  | 0.305 |  |
|  | Total | 113 | 8.009 |  |  | 1.000 |  |
| **Bray-Curtis** | EnvironType_FrogCategory | 4 | 10.214 | 2.553 | 22.456 | 0.452 | 0.0001 |
|  | Residuals | 109 | 12.394 | 0.114 |  | 0.548 |  |
|  | Total | 113 | 22.608 |  |  | 1.000 |  |

**Table S4. Pairwise comparisons of microbial community structure among frog and environment sample types.** Pairwise permutational multivariate analysis of variance (PERMANOVA) model outputs based on unweighted UniFrac distances, weighted UniFrac distances, and Bray-Curtis dissimilarities for frog, rock perch, tank wall, tank water, and underwater rock samples are shown. P-values were adjusted using the Benjamini-Hochberg procedure to control the false discovery rate (FDR) with multiple comparisons. “Signif.” indicates significance of adjusted p-values: “***” = p ≤ 0.001, “**” = p ≤ 0.01, “*” = p ≤ 0.05.

|  | **Combination** | **Sum of Squares** | **Mean Squares** | **F Model** | **R^2^** | **P-value** | **Adjust.  P-value** | **Signif** |
| --- | --- | --- | --- | --- | --- | --- | --- | --- |
| **Unweighted UniFrac** | frog ↔ rock perch | 2.101 | 2.101 | 10.850 | 0.103 | 0.0001 | 0.0003 | *** |
|  | frog ↔ tank wall | 3.470 | 3.470 | 18.187 | 0.158 | 0.0001 | 0.0003 | *** |
|  | frog ↔ tank water | 2.797 | 2.797 | 14.629 | 0.135 | 0.0001 | 0.0003 | *** |
|  | frog ↔ underwater rock | 1.191 | 1.191 | 6.149 | 0.063 | 0.0001 | 0.0003 | *** |
|  | rock perch ↔ tank wall | 0.514 | 0.514 | 2.936 | 0.184 | 0.0002 | 0.0004 | *** |
|  | rock perch ↔ tank water | 0.785 | 0.785 | 4.502 | 0.310 | 0.0025 | 0.0036 | ** |
|  | rock perch ↔ underwater rock | 0.412 | 0.412 | 2.061 | 0.227 | 0.0125 | 0.0125 | * |
|  | tank wall ↔ tank water | 0.659 | 0.659 | 4.179 | 0.243 | 0.0003 | 0.0005 | *** |
|  | tank wall ↔ underwater rock | 0.491 | 0.491 | 2.880 | 0.224 | 0.0053 | 0.0066 | ** |
|  | tank water ↔ underwater rock | 0.507 | 0.507 | 3.032 | 0.302 | 0.0118 | 0.0125 | * |
| **Weighted UniFrac** | frog ↔ rock perch | 1.678 | 1.678 | 80.771 | 0.462 | 0.0001 | 0.0003 | *** |
|  | frog ↔ tank wall | 1.791 | 1.791 | 95.433 | 0.496 | 0.0001 | 0.0003 | *** |
|  | frog ↔ tank water | 1.757 | 1.757 | 96.948 | 0.508 | 0.0001 | 0.0003 | *** |
|  | frog ↔ underwater rock | 1.093 | 1.093 | 62.537 | 0.407 | 0.0001 | 0.0003 | *** |
|  | rock perch ↔ tank wall | 0.399 | 0.399 | 7.493 | 0.366 | 0.0002 | 0.0004 | *** |
|  | rock perch ↔ tank water | 0.346 | 0.346 | 6.019 | 0.376 | 0.0025 | 0.0036 | ** |
|  | rock perch ↔ underwater rock | 0.176 | 0.176 | 2.664 | 0.276 | 0.0352 | 0.0352 | * |
|  | tank wall ↔ tank water | 0.411 | 0.411 | 12.082 | 0.482 | 0.0003 | 0.0005 | *** |
|  | tank wall ↔ underwater rock | 0.420 | 0.420 | 12.748 | 0.560 | 0.0053 | 0.0066 | ** |
|  | tank water ↔ underwater rock | 0.287 | 0.287 | 9.429 | 0.574 | 0.0118 | 0.0131 | * |
| **Bray-Curtis** | frog ↔ rock perch | 2.786 | 2.786 | 27.980 | 0.229 | 0.0001 | 0.0003 | *** |
|  | frog ↔ tank wall | 3.529 | 3.529 | 37.594 | 0.279 | 0.0001 | 0.0003 | *** |
|  | frog ↔ tank water | 3.123 | 3.123 | 33.940 | 0.265 | 0.0001 | 0.0003 | *** |
|  | frog ↔ underwater rock | 1.867 | 1.867 | 20.802 | 0.186 | 0.0001 | 0.0003 | *** |
|  | rock perch ↔ tank wall | 1.058 | 1.058 | 4.288 | 0.248 | 0.0002 | 0.0004 | *** |
|  | rock perch ↔ tank water | 1.132 | 1.132 | 4.116 | 0.292 | 0.0025 | 0.0036 | ** |
|  | rock perch ↔ underwater rock | 0.792 | 0.792 | 2.444 | 0.259 | 0.0125 | 0.0125 | * |
|  | tank wall ↔ tank water | 1.087 | 1.087 | 5.661 | 0.303 | 0.0003 | 0.0005 | *** |
|  | tank wall ↔ underwater rock | 1.080 | 1.080 | 5.364 | 0.349 | 0.0053 | 0.0066 | ** |
|  | tank water ↔ underwater rock | 0.922 | 0.922 | 4.141 | 0.372 | 0.0118 | 0.0125 | * |

**Table S5. Microbial community dispersion among frog and environmental sample types.** Results of permutation tests for homogeneity of multivariate dispersions based on unweighted UniFrac, weighted UniFrac, and Bray-Curtis dissimilarities for frog, rock perch, tank wall, tank water, and underwater rock samples (*i.e.,* “EnvironType_FrogCategory” groupings) are shown. “ns” = not significant, “*” = p ≤ 0.05, “**” = p ≤ 0.01, and “***” = p ≤ 0.001.

|  | **Factor** | **Df** | **Sum of Squares** | **Mean Squares** | **F** | **No. Perm** | **Pr(>F)** |
| --- | --- | --- | --- | --- | --- | --- | --- |
| **Unweighted UniFrac Dispersion** | Groups (EnvironType_FrogCategory) | 4 | 0.056 | 0.014 | 1.709 | 9999 | 0.1583 ns |
|  | Residuals | 109 | 0.890 | 0.008 |  |  |  |
| **Weighted UniFrac Dispersion** | Groups (EnvironType_FrogCategory) | 4 | 0.136 | 0.034 | 12.755 | 9999 | 0.0001  *** |
|  | Residuals | 109 | 0.290 | 0.003 |  |  |  |
| **Bray-Curtis Dispersion** | Groups (EnvironType_FrogCategory) | 4 | 0.638 | 0.159 | 12.443 | 9999 | 0.0001  *** |
|  | Residuals | 109 | 1.397 | 0.013 |  |  |  |

**Table S6. Pairwise comparisons of microbial community dispersion among frog and environmental sample types.** Results of *post hoc* Tukey multiple comparisons of mean dispersions based on weighted UniFrac and Bray-Curtis dissimilarities for frog, rock perch, tank wall, tank water, and underwater rock samples are shown. P-values were adjusted using Benjamini-Hochberg procedure to control the false discovery rate (FDR) with multiple comparisons. “Signif.” indicates significance of adjusted p-values: “ns” = not significant, “*” = p ≤ 0.05, “**” = p ≤ 0.01**,** “***” = p ≤ 0.001.

|  | **Combination** | **Difference** | **Lower 95% CI** | **Upper 95% CI** | **Adjusted  P-value** | **Signif.** |
| --- | --- | --- | --- | --- | --- | --- |
| **Weighted UniFrac Dispersion** | rock perch↔frog | 0.142 | 0.082 | 0.202 | 0.00000002 | *** |
|  | tank wall↔frog | 0.054 | 0.004 | 0.104 | 0.02957302 | * |
|  | tank water↔frog | 0.044 | -0.016 | 0.105 | 0.25749055 | ns |
|  | underwater rock↔frog | 0.008 | -0.076 | 0.091 | 0.99914303 | ns |
|  | tank wall↔rock perch | -0.089 | -0.164 | -0.013 | 0.01278466 | * |
|  | tank water↔rock perch | -0.098 | -0.180 | -0.015 | 0.01168291 | * |
|  | underwater rock↔rock perch | -0.135 | -0.236 | -0.033 | 0.00318957 | ** |
|  | tank water↔tank wall | -0.009 | -0.085 | 0.066 | 0.99697405 | ns |
|  | underwater rock↔tank wall | -0.046 | -0.141 | 0.049 | 0.66816610 | ns |
|  | underwater rock↔tank water | -0.037 | -0.138 | 0.064 | 0.85225254 | ns |
| **Bray-Curtis Dispersion** | rock perch↔frog | 0.270 | 0.137 | 0.402 | 0.00000128 | *** |
|  | tank wall↔frog | 0.133 | 0.023 | 0.243 | 0.00949130 | ** |
|  | tank water↔frog | 0.145 | 0.012 | 0.277 | 0.02476239 | * |
|  | underwater rock↔frog | 0.149 | -0.035 | 0.333 | 0.17145704 | ns |
|  | tank wall↔rock perch | -0.137 | -0.302 | 0.029 | 0.15428667 | ns |
|  | tank water↔rock perch | -0.125 | -0.306 | 0.056 | 0.31732904 | ns |
|  | underwater rock↔rock perch | -0.121 | -0.343 | 0.101 | 0.56071676 | ns |
|  | tank water↔tank wall | 0.012 | -0.154 | 0.178 | 0.99963007 | ns |
|  | underwater rock↔tank wall | 0.016 | -0.193 | 0.226 | 0.99949948 | ns |
|  | underwater rock↔tank water | 0.004 | -0.218 | 0.226 | 0.99999793 | ns |

**Table S7. PERMANOVA models for frog microbiome structure.** Permutational multivariate analysis of variance (PERMANOVA) sequential model outputs based on unweighted UniFrac, weighted UniFrac, and Bray-Curtis dissimilarities for frog samples are shown. Bold R^2^ values for body region show that these values do not change after accounting for variation associated with other factors. For significance, “ns” = not significant, “*” = p ≤ 0.05, “**” = p ≤ 0.01, “***” = p ≤ 0.001.

|  | **Model** | **Factor** | **Df** | **Sums Of Squares** | **Mean Squares** | **F Model** | **R^2^** | **Pr(>F)** | **Significance** |
| --- | --- | --- | --- | --- | --- | --- | --- | --- | --- |
| **Unweighted  UniFrac** | **1** | Body Region | 9 | 1.811 | 0.201 | 1.253 | **0.105** | 0.0440 | * |
|  |  | Individual | 8 | 3.844 | 0.481 | 2.993 | 0.223 | 0.0001 | *** |
|  |  | Residuals | 72 | 11.561 | 0.161 |  | 0.672 |  |  |
|  |  | Total | 89 | 17.216 |  |  | 1.000 |  |  |
|  | **2** | Individual | 8 | 3.844 | 0.481 | 2.993 | 0.223 | 0.0001 | *** |
|  |  | Body Region | 9 | 1.811 | 0.201 | 1.253 | **0.105** | 0.0440 | * |
|  |  | Residuals | 72 | 11.561 | 0.161 |  | 0.672 |  |  |
|  |  | Total | 89 | 17.216 |  |  | 1.000 |  |  |
|  | **3** | Tank ID | 2 | 1.067 | 0.533 | 3.321 | 0.062 | 0.0001 | *** |
|  |  | Individual | 6 | 2.777 | 0.463 | 2.883 | 0.161 | 0.0001 | *** |
|  |  | Body Region | 9 | 1.811 | 0.201 | 1.253 | **0.105** | 0.0440 | * |
|  |  | Residuals | 72 | 11.561 | 0.161 |  | 0.672 |  |  |
|  |  | Total | 89 | 17.216 |  |  | 1.000 |  |  |
| **Weighted  UniFrac** | **1** | Body Region | 9 | 0.431 | 0.048 | 7.091 | **0.279** | 0.0001 | *** |
|  |  | Individual | 8 | 0.625 | 0.078 | 11.574 | 0.405 | 0.0001 | *** |
|  |  | Residuals | 72 | 0.486 | 0.007 |  | 0.315 |  |  |
|  |  | Total | 89 | 1.541 |  |  | 1.000 |  |  |
|  | **2** | Individual | 8 | 0.625 | 0.078 | 11.574 | 0.405 | 0.0001 | *** |
|  |  | Body Region | 9 | 0.431 | 0.048 | 7.091 | **0.279** | 0.0001 | *** |
|  |  | Residuals | 72 | 0.486 | 0.007 |  | 0.315 |  |  |
|  |  | Total | 89 | 1.541 |  |  | 1.000 |  |  |
|  | **3** | Tank ID | 2 | 0.405 | 0.203 | 30.039 | 0.263 | 0.0001 | *** |
|  |  | Individual | 6 | 0.219 | 0.037 | 5.419 | 0.142 | 0.0001 | *** |
|  |  | Body Region | 9 | 0.431 | 0.048 | 7.091 | **0.279** | 0.0001 | *** |
|  |  | Residuals | 72 | 0.486 | 0.007 |  | 0.315 |  |  |
|  |  | Total | 89 | 1.541 |  |  | 1.000 |  |  |
| **Bray-Curtis** | **1** | Body Region | 9 | 2.474 | 0.275 | 8.403 | **0.324** | 0.0001 | *** |
|  |  | Individual | 8 | 2.800 | 0.350 | 10.703 | 0.367 | 0.0001 | *** |
|  |  | Residuals | 72 | 2.355 | 0.033 |  | 0.309 |  |  |
|  |  | Total | 89 | 7.629 |  |  | 1.000 |  |  |
|  | **2** | Individual | 8 | 2.800 | 0.350 | 10.703 | 0.367 | 0.0001 | *** |
|  |  | Body Region | 9 | 2.474 | 0.275 | 8.403 | **0.324** | 0.0001 | *** |
|  |  | Residuals | 72 | 2.355 | 0.033 |  | 0.309 |  |  |
|  |  | Total | 89 | 7.629 |  |  | 1.000 |  |  |
|  | **3** | Tank ID | 2 | 1.558 | 0.779 | 23.815 | 0.204 | 0.0001 | *** |
|  |  | Individual | 6 | 1.243 | 0.207 | 6.333 | 0.163 | 0.0001 | *** |
|  |  | Body Region | 9 | 2.474 | 0.275 | 8.403 | **0.324** | 0.0001 | *** |
|  |  | Residuals | 72 | 2.355 | 0.033 |  | 0.309 |  |  |
|  |  | Total | 89 | 7.629 |  |  | 1.000 |  |  |

**Table S8. Pairwise comparisons of microbial community structure among frog body regions.** Pairwise permutational multivariate analysis of variance (PERMANOVA) model outputs based on unweighted UniFrac distances, weighted UniFrac distances, and Bray-Curtis dissimilarities for frog body regions are shown. P-values were adjusted using the Benjamini-Hochberg procedure to control the false discovery rate (FDR) with multiple comparisons. “Signif.” indicates significance of adjusted p-values: “***” = p ≤ 0.001, “**” = p ≤ 0.01, “*” = p ≤ 0.05, “ns” = not significant.

|  | **Combination** | **Sums of Squares** | **Mean Squares** | **F Model** | **R^2^** | **P-value** | **Adjust.  P-value** | **Signif.** |
| --- | --- | --- | --- | --- | --- | --- | --- | --- |
| **Unweighted  UniFrac** | abdomen ↔ back | 0.435 | 0.435 | 2.145 | 0.118 | 0.015 | 0.693 | ns |
|  | abdomen ↔ cloaca | 0.188 | 0.188 | 1.164 | 0.068 | 0.284 | 0.697 | ns |
|  | abdomen ↔ forefeet | 0.285 | 0.285 | 1.727 | 0.097 | 0.062 | 0.697 | ns |
|  | abdomen ↔ hindfeet | 0.204 | 0.204 | 1.297 | 0.075 | 0.217 | 0.697 | ns |
|  | abdomen ↔ inner forelimbs | 0.175 | 0.175 | 1.107 | 0.065 | 0.336 | 0.697 | ns |
|  | abdomen ↔ inner hindlimbs | 0.139 | 0.139 | 0.790 | 0.047 | 0.674 | 0.919 | ns |
|  | abdomen ↔ outer hindlimbs | 0.260 | 0.260 | 1.365 | 0.079 | 0.180 | 0.697 | ns |
|  | abdomen ↔ snout | 0.286 | 0.286 | 1.529 | 0.087 | 0.091 | 0.697 | ns |
|  | abdomen ↔ vocal sack | 0.259 | 0.259 | 1.511 | 0.086 | 0.098 | 0.697 | ns |
|  | back ↔ cloaca | 0.247 | 0.247 | 1.163 | 0.068 | 0.288 | 0.697 | ns |
|  | back ↔ forefeet | 0.235 | 0.235 | 1.087 | 0.064 | 0.334 | 0.697 | ns |
|  | back ↔ hindfeet | 0.231 | 0.231 | 1.108 | 0.065 | 0.317 | 0.697 | ns |
|  | back ↔ inner forelimbs | 0.279 | 0.279 | 1.334 | 0.077 | 0.185 | 0.697 | ns |
|  | back ↔ inner hindlimbs | 0.249 | 0.249 | 1.095 | 0.064 | 0.328 | 0.697 | ns |
|  | back ↔ outer hindlimbs | 0.265 | 0.265 | 1.097 | 0.064 | 0.337 | 0.697 | ns |
|  | back ↔ snout | 0.117 | 0.117 | 0.492 | 0.030 | 0.966 | 0.966 | ns |
|  | back ↔ vocal sack | 0.270 | 0.270 | 1.212 | 0.070 | 0.241 | 0.697 | ns |
|  | cloaca ↔ forefeet | 0.119 | 0.119 | 0.682 | 0.041 | 0.799 | 0.946 | ns |
|  | cloaca ↔ hindfeet | 0.113 | 0.113 | 0.678 | 0.041 | 0.772 | 0.946 | ns |
|  | cloaca ↔ inner forelimbs | 0.064 | 0.064 | 0.381 | 0.023 | 0.956 | 0.966 | ns |
|  | cloaca ↔ inner hindlimbs | 0.169 | 0.169 | 0.912 | 0.054 | 0.514 | 0.826 | ns |
|  | cloaca ↔ outer hindlimbs | 0.301 | 0.301 | 1.505 | 0.086 | 0.126 | 0.697 | ns |
|  | cloaca ↔ snout | 0.121 | 0.121 | 0.616 | 0.037 | 0.885 | 0.966 | ns |
|  | cloaca ↔ vocal sack | 0.140 | 0.140 | 0.774 | 0.046 | 0.694 | 0.919 | ns |
|  | forefeet ↔ hindfeet | 0.096 | 0.096 | 0.565 | 0.034 | 0.915 | 0.966 | ns |
|  | forefeet ↔ inner forelimbs | 0.140 | 0.140 | 0.815 | 0.048 | 0.649 | 0.912 | ns |
|  | forefeet ↔ inner hindlimbs | 0.225 | 0.225 | 1.186 | 0.069 | 0.273 | 0.697 | ns |
|  | forefeet ↔ outer hindlimbs | 0.379 | 0.379 | 1.860 | 0.104 | 0.052 | 0.697 | ns |
|  | forefeet ↔ snout | 0.110 | 0.110 | 0.551 | 0.033 | 0.942 | 0.966 | ns |
|  | forefeet ↔ vocal sack | 0.178 | 0.178 | 0.964 | 0.057 | 0.497 | 0.826 | ns |
|  | hindfeet ↔ inner forelimbs | 0.175 | 0.175 | 1.071 | 0.063 | 0.379 | 0.741 | ns |
|  | hindfeet ↔ inner hindlimbs | 0.151 | 0.151 | 0.828 | 0.049 | 0.590 | 0.912 | ns |
|  | hindfeet ↔ outer hindlimbs | 0.280 | 0.280 | 1.428 | 0.082 | 0.157 | 0.697 | ns |
|  | hindfeet ↔ snout | 0.098 | 0.098 | 0.510 | 0.031 | 0.955 | 0.966 | ns |
|  | hindfeet ↔ vocal sack | 0.146 | 0.146 | 0.829 | 0.049 | 0.635 | 0.912 | ns |
|  | inner forelimbs ↔ inner hindlimbs | 0.128 | 0.128 | 0.700 | 0.042 | 0.765 | 0.946 | ns |
|  | inner forelimbs ↔ outer hindlimbs | 0.304 | 0.304 | 1.542 | 0.088 | 0.122 | 0.697 | ns |
|  | inner forelimbs ↔ snout | 0.182 | 0.182 | 0.939 | 0.055 | 0.505 | 0.826 | ns |
|  | inner forelimbs ↔ vocal sack | 0.183 | 0.183 | 1.031 | 0.061 | 0.419 | 0.785 | ns |
|  | inner hindlimbs ↔ outer hindlimbs | 0.172 | 0.172 | 0.799 | 0.048 | 0.629 | 0.912 | ns |
|  | inner hindlimbs ↔ snout | 0.146 | 0.146 | 0.691 | 0.041 | 0.794 | 0.946 | ns |
|  | inner hindlimbs ↔ vocal sack | 0.191 | 0.191 | 0.973 | 0.057 | 0.484 | 0.826 | ns |
|  | outer hindlimbs ↔ snout | 0.268 | 0.268 | 1.186 | 0.069 | 0.265 | 0.697 | ns |
|  | outer hindlimbs ↔ vocal sack | 0.229 | 0.229 | 1.089 | 0.064 | 0.341 | 0.697 | ns |
|  | snout ↔ vocal sack | 0.123 | 0.123 | 0.596 | 0.036 | 0.934 | 0.966 | ns |
| **Weighted  UniFrac** | abdomen ↔ back | 0.117 | 0.117 | 8.998 | 0.360 | 0.003 | 0.019 | * |
|  | abdomen ↔ cloaca | 0.009 | 0.009 | 0.613 | 0.037 | 0.541 | 0.652 | ns |
|  | abdomen ↔ forefeet | 0.053 | 0.053 | 3.193 | 0.166 | 0.055 | 0.145 | ns |
|  | abdomen ↔ hindfeet | 0.008 | 0.008 | 0.595 | 0.036 | 0.462 | 0.630 | ns |
|  | abdomen ↔ inner forelimbs | 0.010 | 0.010 | 0.613 | 0.037 | 0.499 | 0.641 | ns |
|  | abdomen ↔ inner hindlimbs | 0.004 | 0.004 | 0.267 | 0.016 | 0.707 | 0.723 | ns |
|  | abdomen ↔ outer hindlimbs | 0.007 | 0.007 | 0.594 | 0.036 | 0.522 | 0.652 | ns |
|  | abdomen ↔ snout | 0.072 | 0.072 | 4.068 | 0.203 | 0.025 | 0.081 | ns |
|  | abdomen ↔ vocal sack | 0.026 | 0.026 | 1.433 | 0.082 | 0.229 | 0.368 | ns |
|  | back ↔ cloaca | 0.160 | 0.160 | 14.861 | 0.482 | 0.000 | 0.007 | ** |
|  | back ↔ forefeet | 0.177 | 0.177 | 13.637 | 0.460 | 0.001 | 0.008 | ** |
|  | back ↔ hindfeet | 0.184 | 0.184 | 19.896 | 0.554 | 0.000 | 0.007 | ** |
|  | back ↔ inner forelimbs | 0.103 | 0.103 | 8.172 | 0.338 | 0.002 | 0.015 | * |
|  | back ↔ inner hindlimbs | 0.138 | 0.138 | 12.648 | 0.441 | 0.001 | 0.011 | * |
|  | back ↔ outer hindlimbs | 0.106 | 0.106 | 12.131 | 0.431 | 0.001 | 0.011 | * |
|  | back ↔ snout | 0.022 | 0.022 | 1.581 | 0.090 | 0.172 | 0.322 | ns |
|  | back ↔ vocal sack | 0.064 | 0.064 | 4.407 | 0.216 | 0.012 | 0.055 | ns |
|  | cloaca ↔ forefeet | 0.023 | 0.023 | 1.568 | 0.089 | 0.222 | 0.368 | ns |
|  | cloaca ↔ hindfeet | 0.005 | 0.005 | 0.514 | 0.031 | 0.627 | 0.674 | ns |
|  | cloaca ↔ inner forelimbs | 0.010 | 0.010 | 0.682 | 0.041 | 0.492 | 0.641 | ns |
|  | cloaca ↔ inner hindlimbs | 0.002 | 0.002 | 0.197 | 0.012 | 0.882 | 0.882 | ns |
|  | cloaca ↔ outer hindlimbs | 0.009 | 0.009 | 0.891 | 0.053 | 0.413 | 0.599 | ns |
|  | cloaca ↔ snout | 0.085 | 0.085 | 5.558 | 0.258 | 0.008 | 0.040 | * |
|  | cloaca ↔ vocal sack | 0.030 | 0.030 | 1.884 | 0.105 | 0.155 | 0.302 | ns |
|  | forefeet ↔ hindfeet | 0.050 | 0.050 | 3.881 | 0.195 | 0.031 | 0.094 | ns |
|  | forefeet ↔ inner forelimbs | 0.022 | 0.022 | 1.357 | 0.078 | 0.256 | 0.397 | ns |
|  | forefeet ↔ inner hindlimbs | 0.032 | 0.032 | 2.229 | 0.122 | 0.117 | 0.264 | ns |
|  | forefeet ↔ outer hindlimbs | 0.036 | 0.036 | 2.920 | 0.154 | 0.067 | 0.167 | ns |
|  | forefeet ↔ snout | 0.081 | 0.081 | 4.615 | 0.224 | 0.022 | 0.075 | ns |
|  | forefeet ↔ vocal sack | 0.036 | 0.036 | 1.974 | 0.110 | 0.147 | 0.300 | ns |
|  | hindfeet ↔ inner forelimbs | 0.022 | 0.022 | 1.790 | 0.101 | 0.186 | 0.335 | ns |
|  | hindfeet ↔ inner hindlimbs | 0.006 | 0.006 | 0.515 | 0.031 | 0.550 | 0.652 | ns |
|  | hindfeet ↔ outer hindlimbs | 0.018 | 0.018 | 2.054 | 0.114 | 0.138 | 0.295 | ns |
|  | hindfeet ↔ snout | 0.117 | 0.117 | 8.477 | 0.346 | 0.002 | 0.015 | * |
|  | hindfeet ↔ vocal sack | 0.052 | 0.052 | 3.566 | 0.182 | 0.044 | 0.125 | ns |
|  | inner forelimbs ↔ inner hindlimbs | 0.006 | 0.006 | 0.453 | 0.028 | 0.591 | 0.665 | ns |
|  | inner forelimbs ↔ outer hindlimbs | 0.007 | 0.007 | 0.551 | 0.033 | 0.568 | 0.655 | ns |
|  | inner forelimbs ↔ snout | 0.044 | 0.044 | 2.582 | 0.139 | 0.093 | 0.220 | ns |
|  | inner forelimbs ↔ vocal sack | 0.007 | 0.007 | 0.394 | 0.024 | 0.666 | 0.697 | ns |
|  | inner hindlimbs ↔ outer hindlimbs | 0.005 | 0.005 | 0.461 | 0.028 | 0.629 | 0.674 | ns |
|  | inner hindlimbs ↔ snout | 0.077 | 0.077 | 4.980 | 0.237 | 0.016 | 0.065 | ns |
|  | inner hindlimbs ↔ vocal sack | 0.024 | 0.024 | 1.480 | 0.085 | 0.225 | 0.368 | ns |
|  | outer hindlimbs ↔ snout | 0.053 | 0.053 | 3.989 | 0.200 | 0.021 | 0.075 | ns |
|  | outer hindlimbs ↔ vocal sack | 0.016 | 0.016 | 1.150 | 0.067 | 0.310 | 0.464 | ns |
|  | snout ↔ vocal sack | 0.016 | 0.016 | 0.839 | 0.050 | 0.445 | 0.625 | ns |
| **Bray-Curtis** | abdomen ↔ back | 0.682 | 0.682 | 10.461 | 0.395 | 0.004 | 0.017 | * |
|  | abdomen ↔ cloaca | 0.036 | 0.036 | 0.471 | 0.029 | 0.639 | 0.670 | ns |
|  | abdomen ↔ forefeet | 0.202 | 0.202 | 2.343 | 0.128 | 0.112 | 0.213 | ns |
|  | abdomen ↔ hindfeet | 0.049 | 0.049 | 0.697 | 0.042 | 0.424 | 0.564 | ns |
|  | abdomen ↔ inner forelimbs | 0.054 | 0.054 | 0.632 | 0.038 | 0.479 | 0.599 | ns |
|  | abdomen ↔ inner hindlimbs | 0.017 | 0.017 | 0.217 | 0.013 | 0.734 | 0.751 | ns |
|  | abdomen ↔ outer hindlimbs | 0.024 | 0.024 | 0.373 | 0.023 | 0.641 | 0.670 | ns |
|  | abdomen ↔ snout | 0.501 | 0.501 | 5.808 | 0.266 | 0.012 | 0.043 | * |
|  | abdomen ↔ vocal sack | 0.190 | 0.190 | 1.961 | 0.109 | 0.153 | 0.266 | ns |
|  | back ↔ cloaca | 0.867 | 0.867 | 19.843 | 0.554 | 0.000 | 0.004 | ** |
|  | back ↔ forefeet | 0.717 | 0.717 | 13.167 | 0.451 | 0.001 | 0.006 | ** |
|  | back ↔ hindfeet | 1.098 | 1.098 | 28.898 | 0.644 | 0.000 | 0.004 | ** |
|  | back ↔ inner forelimbs | 0.549 | 0.549 | 10.347 | 0.393 | 0.002 | 0.010 | ** |
|  | back ↔ inner hindlimbs | 0.794 | 0.794 | 16.407 | 0.506 | 0.001 | 0.006 | ** |
|  | back ↔ outer hindlimbs | 0.655 | 0.655 | 19.525 | 0.550 | 0.000 | 0.004 | ** |
|  | back ↔ snout | 0.075 | 0.075 | 1.383 | 0.080 | 0.244 | 0.379 | ns |
|  | back ↔ vocal sack | 0.269 | 0.269 | 4.139 | 0.206 | 0.019 | 0.056 | ns |
|  | cloaca ↔ forefeet | 0.103 | 0.103 | 1.590 | 0.090 | 0.203 | 0.338 | ns |
|  | cloaca ↔ hindfeet | 0.035 | 0.035 | 0.726 | 0.043 | 0.477 | 0.599 | ns |
|  | cloaca ↔ inner forelimbs | 0.043 | 0.043 | 0.675 | 0.040 | 0.520 | 0.616 | ns |
|  | cloaca ↔ inner hindlimbs | 0.015 | 0.015 | 0.249 | 0.015 | 0.860 | 0.860 | ns |
|  | cloaca ↔ outer hindlimbs | 0.029 | 0.029 | 0.668 | 0.040 | 0.569 | 0.640 | ns |
|  | cloaca ↔ snout | 0.584 | 0.584 | 9.010 | 0.360 | 0.000 | 0.004 | ** |
|  | cloaca ↔ vocal sack | 0.221 | 0.221 | 2.936 | 0.155 | 0.058 | 0.145 | ns |
|  | forefeet ↔ hindfeet | 0.269 | 0.269 | 4.553 | 0.222 | 0.025 | 0.067 | ns |
|  | forefeet ↔ inner forelimbs | 0.050 | 0.050 | 0.681 | 0.041 | 0.502 | 0.610 | ns |
|  | forefeet ↔ inner hindlimbs | 0.157 | 0.157 | 2.267 | 0.124 | 0.114 | 0.213 | ns |
|  | forefeet ↔ outer hindlimbs | 0.129 | 0.129 | 2.368 | 0.129 | 0.094 | 0.208 | ns |
|  | forefeet ↔ snout | 0.362 | 0.362 | 4.786 | 0.230 | 0.012 | 0.043 | * |
|  | forefeet ↔ vocal sack | 0.133 | 0.133 | 1.542 | 0.088 | 0.210 | 0.338 | ns |
|  | hindfeet ↔ inner forelimbs | 0.151 | 0.151 | 2.616 | 0.141 | 0.102 | 0.208 | ns |
|  | hindfeet ↔ inner hindlimbs | 0.042 | 0.042 | 0.784 | 0.047 | 0.400 | 0.563 | ns |
|  | hindfeet ↔ outer hindlimbs | 0.086 | 0.086 | 2.255 | 0.124 | 0.124 | 0.223 | ns |
|  | hindfeet ↔ snout | 0.836 | 0.836 | 14.151 | 0.469 | 0.000 | 0.004 | ** |
|  | hindfeet ↔ vocal sack | 0.389 | 0.389 | 5.597 | 0.259 | 0.016 | 0.051 | ns |
|  | inner forelimbs ↔ inner hindlimbs | 0.053 | 0.053 | 0.779 | 0.046 | 0.426 | 0.564 | ns |
|  | inner forelimbs ↔ outer hindlimbs | 0.031 | 0.031 | 0.575 | 0.035 | 0.555 | 0.640 | ns |
|  | inner forelimbs ↔ snout | 0.319 | 0.319 | 4.306 | 0.212 | 0.020 | 0.056 | ns |
|  | inner forelimbs ↔ vocal sack | 0.082 | 0.082 | 0.973 | 0.057 | 0.370 | 0.537 | ns |
|  | inner hindlimbs ↔ outer hindlimbs | 0.021 | 0.021 | 0.433 | 0.026 | 0.623 | 0.670 | ns |
|  | inner hindlimbs ↔ snout | 0.558 | 0.558 | 8.029 | 0.334 | 0.004 | 0.017 | * |
|  | inner hindlimbs ↔ vocal sack | 0.197 | 0.197 | 2.458 | 0.133 | 0.099 | 0.208 | ns |
|  | outer hindlimbs ↔ snout | 0.448 | 0.448 | 8.189 | 0.339 | 0.001 | 0.006 | ** |
|  | outer hindlimbs ↔ vocal sack | 0.146 | 0.146 | 2.234 | 0.123 | 0.102 | 0.208 | ns |
|  | snout ↔ vocal sack | 0.102 | 0.102 | 1.189 | 0.069 | 0.345 | 0.517 | ns |

**Table S9. Microbial community dispersion by frog body region.** Results of permutation tests for homogeneity of multivariate dispersions based on unweighted UniFrac, weighted UniFrac, and Bray-Curtis dissimilarities for frog body regions are shown. “Signif.” indicates significance p-values: “***” = p ≤ 0.001, “**” = p ≤ 0.01, “*” = p ≤ 0.05, “ns” = not significant.

|  | **Factor** | **Df** | **Sum of Squares** | **Mean Squares** | **F** | **No. Permutations** | **Pr(>F)** | **Signif.** |
| --- | --- | --- | --- | --- | --- | --- | --- | --- |
| **Unweighted UniFrac Dispersion** | Body Region | 9 | 0.110 | 0.012 | 1.369 | 9999 | 0.211 | ns |
|  | Residuals | 80 | 0.715 | 0.009 |  |  |  |  |
| **Weighted UniFrac Dispersion** | Body Region | 9 | 0.030 | 0.003 | 0.820 | 9999 | 0.605 | ns |
|  | Residuals | 80 | 0.321 | 0.004 |  |  |  |  |
| **Bray-Curtis Dispersion** | Body Region | 9 | 0.185 | 0.021 | 1.191 | 9999 | 0.311 | ns |
|  | Residuals | 80 | 1.378 | 0.017 |  |  |  |  |

**Table S10. Pairwise comparisons of relative abundance of the family Burkholderiaceae among frog and environmental sample types.** Following significant results of Kruskal-Wallis test (Burkholderiaceae relative abundance: chi-squared = 357.31, df = 4, p < 0.001), results of *post hoc* Dunn tests for significant differences in relative abundance of the family Burkholderiaceae in pairwise comparisons of frog, rock perch, tank wall, tank water, and underwater rock samples are shown. P-values were adjusted using Benjamini-Hochberg procedure to control the false discovery rate (FDR) with multiple comparisons. For significance, “***” = p ≤ 0.001, “**” = p ≤ 0.01, “*” = p ≤ 0.05, and “ns” = not significant.

| **Comparison** | **Z** | **Unadjusted  P-value** | **Adjusted  P-value** | **Significance** |
| --- | --- | --- | --- | --- |
| frog ↔ rock perch | -14.531 | 7.73E-48 | 7.73E-47 | *** |
| frog ↔ tank wall | -7.349 | 1.99E-13 | 6.65E-13 | *** |
| rock perch ↔ tank wall | 6.750 | 1.48E-11 | 2.96E-11 | *** |
| frog ↔ tank water | -9.939 | 2.83E-23 | 1.41E-22 | *** |
| rock perch ↔ tank water | 3.354 | 7.98E-04 | 1.33E-03 | ** |
| tank wall ↔ tank water | -3.076 | 2.10E-03 | 3.00E-03 | ** |
| frog ↔ underwater rock | -6.984 | 2.87E-12 | 7.18E-12 | *** |
| rock perch ↔ underwater rock | 2.868 | 4.13E-03 | 5.16E-03 | ** |
| tank wall ↔ underwater rock | -2.294 | 2.18E-02 | 2.42E-02 | * |
| tank water ↔ underwater rock | 0.130 | 8.97E-01 | 8.97E-01 | ns |

**Table S11. Pairwise comparisons of relative abundances of amplicon sequence variants among frog and environmental sample types.** Following significant results of Kruskal-Wallis tests (SV2 relative abundance: chi-squared = 56.492, df = 4, p < 0.001; SV1 relative abundance: chi-squared = 45.626, df = 4, p < 0.001), results of *post hoc* Dunn tests for significant differences in relative abundance of SV1 (family Rubritaleaceae) and SV2 (family Burkholderiaceae) in pairwise comparisons of frog, rock perch, tank wall, tank water, and underwater rock samples are shown. P-values were adjusted using Benjamini-Hochberg procedure to control the false discovery rate (FDR) with multiple comparisons. For significance, “***” = p ≤ 0.001, “**” = p ≤ 0.01, “*” = p ≤ 0.05, and “ns” = not significant.

|  | **Comparison** | **Z** | **Unadjusted P-value** | **Adjusted P-value** | **Significance** |
| --- | --- | --- | --- | --- | --- |
| **SV2** | frog ↔ rock perch | 4.335 | 0.000015 | 0.000073 | *** |
|  | frog ↔ tank wall | 4.736 | 0.000002 | 0.000022 | *** |
|  | rock perch ↔ tank wall | -0.327 | 0.743755 | 1.000000 | ns |
|  | frog ↔ tank water | 3.982 | 0.000068 | 0.000227 | *** |
|  | rock perch ↔ tank water | -0.258 | 0.796680 | 1.000000 | ns |
|  | tank wall ↔ tank water | 0.045 | 0.964388 | 1.000000 | ns |
|  | frog ↔ underwater rock | 3.093 | 0.001981 | 0.004953 | ** |
|  | rock perch ↔ underwater rock | -0.018 | 0.985776 | 0.985776 | ns |
|  | tank wall ↔ underwater rock | 0.240 | 0.810706 | 1.000000 | ns |
|  | tank water ↔ underwater rock | 0.193 | 0.847320 | 1.000000 | ns |
| **SV1** | frog ↔ rock perch | 4.233 | 0.000023 | 0.000230 | *** |
|  | frog ↔ tank wall | 3.459 | 0.000543 | 0.001809 | ** |
|  | rock perch ↔ tank wall | -1.092 | 0.274712 | 0.457854 | ns |
|  | frog ↔ tank water | 3.575 | 0.000350 | 0.001748 | ** |
|  | rock perch ↔ tank water | -0.480 | 0.630973 | 0.701081 | ns |
|  | tank wall ↔ tank water | 0.566 | 0.571346 | 0.714183 | ns |
|  | frog ↔ underwater rock | 3.320 | 0.000899 | 0.002247 | ** |
|  | rock perch ↔ underwater rock | 0.232 | 0.816724 | 0.816724 | ns |
|  | tank wall ↔ underwater rock | 1.109 | 0.267284 | 0.534568 | ns |
|  | tank water ↔ underwater rock | 0.624 | 0.532647 | 0.760924 | ns |

**Table S12. Differentially abundant amplicon sequence variants between ventral body regions and the dorsal back region.** Differentially abundant amplicon sequence variants identified by DESeq2 (*i.e.,* those that had significant log_2_ fold difference with p ≤ 0.05) are shown for each contrast. Contrasts examined include abdomen – back, inner hindlimbs – back, forefeet – back, and hindfeet – back. “ASV” indicates the differentially abundant amplicon sequence variant and its associated taxonomy. The "base Mean'' is the mean DESeq2 normalized counts for the amplicon sequence variant across all samples. The "Estimate" is the log_2_ fold difference estimate for the specified contrast. The "Statistic" is the Wald statistic for the specified contrast. P-values are adjusted for multiple comparisons using the Benjamini-Hochberg procedure. “Signif.” indicates significance of adjusted p-values: “***” = p ≤ 0.001, “**” = p ≤ 0.01, “*” = p ≤ 0.05, “ns” = not significant.

| **Contrast** | **ASV** | **base Mean** | **Estimate** | **SE** | **Statistic** | **P-value** | **Adjust.  P-value** | **Signif.** |
| --- | --- | --- | --- | --- | --- | --- | --- | --- |
| abdomen ↔ back | SV56 F. Burkholderiaceae | 10.237 | 24.127 | 6.488 | 3.719 | 0.0002 | 0.0498 | * |

**Table S13. Taxonomy of putative *Bd*-inhibitory community members.** Assigned taxonomy (SILVA v. 132) for all 73 frog-associated ASVs that shared ≥ 99% identity with sequences from a database of bacterial isolates shown to inhibit *Batrachochytrium* pathogens (Woodhams et al., 2015). Asterisk (*) indicates that the ASV shared 100% identity with an inhibitory database sequence. This strict subset (29 ASVs) was used to calculate putative *Bd*-inhibitory relative abundance.

| **Phylum** | **Family** | **Genus** | **Species** | **ASV** |
| --- | --- | --- | --- | --- |
| **Actinobacteria** | Microbacteriaceae | Microbacterium | Unclassified | SV137*, SV347 |
|  | Nocardiaceae | Rhodococcus | Unclassified | SV1465 |
|  | Sanguibacteraceae | Sanguibacter | Unclassified | SV1634* |
| **Bacteroidetes** | Flavobacteriaceae | Myroides | odoratus | SV757* |
|  |  |  | Unclassified | SV1508 |
|  | Weeksellaceae | Chryseobacterium | greenlandense | SV22* |
|  |  |  | Unclassified | SV313*, SV1933 |
| **Firmicutes** | Bacillaceae | Bacillus | Unclassified | SV34* |
|  | Staphylococcaceae | Staphylococcus | Unclassified | SV223 |
| **Proteobacteria** | Aeromonadaceae | Aeromonas | Unclassified | SV91*, SV1782*, SV1080 |
|  | Beijerinckiaceae | Bosea | Unclassified | SV109, SV304 |
|  | Burkholderiaceae | Delftia | Unclassified | SV531* |
|  |  | Janthinobacterium | agaricidamnosum | SV1631 |
|  |  |  | lividum | SV1213 |
|  |  |  | Unclassified | SV100, SV105*, SV173* |
|  |  | Unclassified | Unclassified | SV2*, SV56, SV701* |
|  |  | Xenophilus | Unclassified | SV144*, SV1563, SV1845 |
|  | Enterobacteriaceae | Enterobacter | Unclassified | SV1072* |
|  |  | Rahnella | aquatilis | SV497, SV651 |
|  |  |  | Unclassified | SV124, SV248, SV336*, SV615*, SV753, SV1073, SV1431, SV1551 |
|  |  | Serratia | fonticola | SV383* |
|  |  |  | Unclassified | SV1060, SV1241 |
|  |  | Unclassified | Unclassified | SV239, SV416, SV439, SV599, SV892, SV951 |
|  | Pseudomonadaceae | Pseudomonas | arsenicoxydans | SV52 |
|  |  |  | lurida | SV154* |
|  |  |  | Unclassified | SV5*, SV29, SV37, SV38*, SV60*, SV61, SV361*, SV454, SV467*, SV910, SV1178*, SV1254, SV1549, SV1795* |
|  | Rhizobiaceae | Brucella | Unclassified | SV1507* |
|  | Sphingomonadaceae | Sphingomonas | mucosissima | SV286 |
|  |  |  | Unclassified | SV289, SV1596, SV2087 |
|  | Xanthomonadaceae | Stenotrophomonas | Unclassified | SV97*, SV235, SV324, SV2439* |

**Table S14. Pairwise comparisons of putative *Bd*-inhibitory relative abundance among frog body regions.** We calculated relative abundances of putative *Bd-*inhibitory taxa based on our strict subset of 29 frog-associated ASVs that shared 100% sequence identity with inhibitory database taxa (Woodhams et al., 2015). Following significant results of a Kruskal-Wallis test (chi-squared = 34.19, df = 9, p < 0.001), results of *post hoc* Dunn tests are shown. P-values were adjusted using Benjamini-Hochberg procedure. For significance, “***” = p ≤ 0.001, “**” = p ≤ 0.01, “*” = p ≤ 0.05, and “ns” = not significant.

| **Comparison** | **Z** | **Unadjusted  P-value** | **Adjusted  P-value** | **Significance** |
| --- | --- | --- | --- | --- |
| abdomen - back | 3.465 | 0.00053 | 0.00478 | ** |
| abdomen - cloaca | -0.307 | 0.75903 | 0.85391 | ns |
| back - cloaca | -3.771 | 0.00016 | 0.00365 | ** |
| abdomen - forefeet | 0.523 | 0.60077 | 0.73067 | ns |
| back - forefeet | -2.941 | 0.00327 | 0.01634 | * |
| cloaca - forefeet | 0.830 | 0.40651 | 0.65332 | ns |
| abdomen - hindfeet | -0.947 | 0.34347 | 0.57245 | ns |
| back - hindfeet | -4.412 | 0.00001 | 0.00046 | *** |
| cloaca - hindfeet | -0.641 | 0.52180 | 0.67088 | ns |
| forefeet - hindfeet | -1.471 | 0.14139 | 0.31813 | ns |
| abdomen - inner forelimbs | 0.510 | 0.61022 | 0.72263 | ns |
| back - inner forelimbs | -2.955 | 0.00313 | 0.02011 | * |
| cloaca - inner forelimbs | 0.817 | 0.41421 | 0.60127 | ns |
| forefeet - inner forelimbs | -0.014 | 0.98920 | 0.98920 | ns |
| hindfeet - inner forelimbs | 1.457 | 0.14509 | 0.31091 | ns |
| abdomen - inner hindlimbs | -0.131 | 0.89592 | 0.95991 | ns |
| back - inner hindlimbs | -3.595 | 0.00032 | 0.00486 | ** |
| cloaca - inner hindlimbs | 0.176 | 0.86035 | 0.94428 | ns |
| forefeet - inner hindlimbs | -0.654 | 0.51304 | 0.69960 | ns |
| hindfeet - inner hindlimbs | 0.817 | 0.41421 | 0.62131 | ns |
| inner forelimbs - inner hindlimbs | -0.641 | 0.52180 | 0.69061 | ns |
| abdomen - outer hindlimbs | 0.433 | 0.66497 | 0.76727 | ns |
| back - outer hindlimbs | -3.031 | 0.00243 | 0.01825 | * |
| cloaca - outer hindlimbs | 0.740 | 0.45941 | 0.64604 | ns |
| forefeet - outer hindlimbs | -0.090 | 0.92811 | 0.97128 | ns |
| hindfeet - outer hindlimbs | 1.380 | 0.16746 | 0.34254 | ns |
| inner forelimbs - outer hindlimbs | -0.077 | 0.93887 | 0.96021 | ns |
| inner hindlimbs - outer hindlimbs | 0.564 | 0.57283 | 0.71604 | ns |
| abdomen - snout | 2.644 | 0.00821 | 0.03357 | * |
| back - snout | -0.821 | 0.41163 | 0.63874 | ns |
| cloaca - snout | 2.950 | 0.00317 | 0.01786 | * |
| forefeet - snout | 2.120 | 0.03399 | 0.10196 | ns |
| hindfeet - snout | 3.591 | 0.00033 | 0.00371 | ** |
| inner forelimbs - snout | 2.134 | 0.03286 | 0.10563 | ns |
| inner hindlimbs - snout | 2.774 | 0.00553 | 0.02489 | * |
| outer hindlimbs - snout | 2.210 | 0.02707 | 0.09372 | ns |
| abdomen - vocal sack | 1.525 | 0.12732 | 0.30155 | ns |
| back - vocal sack | -1.940 | 0.05241 | 0.14739 | ns |
| cloaca - vocal sack | 1.832 | 0.06702 | 0.17742 | ns |
| forefeet - vocal sack | 1.001 | 0.31660 | 0.54796 | ns |
| hindfeet - vocal sack | 2.472 | 0.01343 | 0.05037 | ns |
| inner forelimbs - vocal sack | 1.015 | 0.31011 | 0.55819 | ns |
| inner hindlimbs - vocal sack | 1.656 | 0.09781 | 0.24452 | ns |
| outer hindlimbs - vocal sack | 1.092 | 0.27497 | 0.51557 | ns |
| snout - vocal sack | -1.119 | 0.26324 | 0.51504 | ns |
